# Cost-effectiveness of integrating postpartum antiretroviral therapy and infant care into maternal & child health services in South Africa

**DOI:** 10.1371/journal.pone.0225104

**Published:** 2019-11-15

**Authors:** Caitlin M. Dugdale, Tamsin K. Phillips, Landon Myer, Emily P. Hyle, Kirsty Brittain, Kenneth A. Freedberg, Lucy Cunnama, Rochelle P. Walensky, Allison Zerbe, Milton C. Weinstein, Elaine J. Abrams, Andrea L. Ciaranello, the MCH-ART Trial Team

**Affiliations:** 1 Medical Practice Evaluation Center, Department of Medicine, Massachusetts General Hospital, Boston, MA, United States of America; 2 Division of Infectious Diseases, Department of Medicine, Massachusetts General Hospital, Boston, MA, United States of America; 3 Harvard Medical School, Boston, MA, United States of America; 4 Division of Epidemiology and Biostatistics, School of Public Health & Family Medicine, University of Cape Town, Cape Town, South Africa; 5 Centre for Infectious Diseases Epidemiology & Research, School of Public Health & Family Medicine, University of Cape Town, Cape Town, South Africa; 6 Division of General Internal Medicine, Massachusetts General Hospital, Boston, MA, United States of America; 7 Department of Health Policy and Management, Harvard T.H. Chan School of Public Health, Boston, MA, United States of America; 8 Health Economics Unit, School of Public Health & Family Medicine, University of Cape Town, South Africa; 9 ICAP at Columbia and the Mailman School of Public Health, Columbia University, New York, NY, United States of America; 10 College of Physicians & Surgeons, Columbia University, New York, NY, United States of America; NPMS-HHC CIC / LSH&TM, UNITED KINGDOM

## Abstract

**Background:**

Poor engagement in postpartum maternal HIV care is a challenge worldwide and contributes to adverse maternal outcomes and vertical transmission. Our objective was to project the clinical and economic impact of integrated postpartum maternal antiretroviral therapy (ART) and pediatric care in South Africa.

**Methods:**

Using the CEPAC computer simulation models, parameterized with data from the Maternal and Child Health–Antiretroviral Therapy (MCH-ART) randomized controlled trial, we evaluated the cost-effectiveness of integrated postpartum care for women initiating ART in pregnancy and their children. We compared two strategies: 1) standard of care (*SOC*) referral to local clinics after delivery for separate standard ART services for women and pediatric care for infants, and 2) the MCH-ART intervention (*MCH-ART*) of co-located maternal/pediatric care integrated in Maternal and Child Health (MCH) services throughout breastfeeding. Trial-derived inputs included: 12-month maternal retention in care and virologic suppression (*SOC*: 49%, *MCH-ART*: 67%), breastfeeding duration (*SOC*: 6 months, *MCH-ART*: 8 months), and postpartum healthcare costs for mother-infant pairs (*SOC*: $50, *MCH-ART*: $69). Outcomes included pediatric HIV infections, maternal and infant life expectancy (LE), lifetime HIV-related per-person costs, and incremental cost-effectiveness ratios (ICERs; ICER <US$903/YLS considered “cost-effective”).

**Results:**

Compared to *SOC*, *MCH-ART* increased maternal LE (*SOC*: 25.26 years, *MCH-ART*: 26.20 years) and lifetime costs (*SOC*: $9,912, *MCH-ART*: $10,207; discounted). Projected pediatric outcomes for all HIV-exposed children were similar between arms, although undiscounted LE for HIV-infected children was shorter in *SOC* (*SOC*: 23.13 years, *MCH-ART*: 23.40 years). Combining discounted maternal and pediatric outcomes, the ICER was $599/YLS.

**Conclusion:**

Co-located maternal HIV and pediatric care, integrated in MCH services throughout breastfeeding, is a cost-effective strategy to improve maternal and pediatric outcomes and should be implemented in South Africa.

## Introduction

The postpartum period is a high-risk time for women with HIV and their children. Despite the scale-up of lifelong antiretroviral therapy (ART) for all pregnant and breastfeeding women, approximately 180,000 new pediatric HIV infections occurred worldwide in 2017, including 13,000 in South Africa [1]. High rates of postpartum loss to follow-up (LTFU) and ART non-adherence contribute to adverse maternal outcomes and HIV transmission to breastfeeding infants [2–4]. By 12 months postpartum, 20–30% of women with HIV are lost to follow-up, and only 75% of those in care are virologically suppressed [2–4]. Postpartum disengagement from care thus represents a substantial obstacle impeding the elimination of pediatric HIV infection and better overall maternal and child health (MCH).

In South Africa, most pregnant women with HIV receive HIV care and ART integrated into antenatal services in MCH clinics [5]. After delivery, HIV care is transferred to separate general ART services for women and routine pediatric care for children; this is a particularly vulnerable time for disengagement [5]. Integrating postpartum ART services for mothers and infant care into MCH services has been proposed to address this vulnerability [5, 6]. However, there are concerns that service integration could overburden providers, leading to reduced quality of care and/or increased downstream costs [6, 7].

To examine these potential tradeoffs, the Maternal and Child Health–Antiretroviral Therapy (MCH-ART) trial evaluated a novel platform of integrated postpartum care in South Africa [8]. The trial enrolled breastfeeding women who had started ART during pregnancy. The current standard of care was compared to the MCH-ART intervention of integrating concurrent and co-located maternal ART and pediatric care into the MCH clinic through the end of breastfeeding. The MCH-ART intervention increased the primary endpoint of combined maternal retention and virologic suppression (HIV RNA <50 copies/mL) at 12 months postpartum and extended breastfeeding duration [8]. Our objective was to project the long-term clinical outcomes and cost-effectiveness of the MCH-ART approach to integrated postpartum care, beyond the 12-month horizon of the randomized trial, for women with HIV and their children in South Africa.

## Methods

### Analytic overview

We used the Cost-effectiveness of Preventing AIDS Complications (CEPAC)-International and CEPAC-Pediatrics models to evaluate two strategies: standard of care (*SOC*) and the MCH-ART intervention (*MCH-ART*) [9–11]. We simulated a cohort of women who initiated ART during pregnancy and a cohort of their breastfed children from delivery through death. Maternal retention and virologic suppression through the first 12 months postpartum were calibrated to match MCH-ART trial results; clinical and economic outcomes for women and children beyond the 12 month trial period were modeled using input data from the published literature (Table 1, S1 Table) [8]. All simulated women and children face monthly risks of non-HIV-related mortality. Individuals with HIV face monthly risks of virologic failure, LTFU, opportunistic diseases (ODs), and HIV-related mortality. Full model specifications are available online [12].

**Table 1:** Selected model input parameters.

Variable	Base case value			Range examined^a^	Sources
A. Maternal cohort characteristics					
Age, mean (SD), years	28.6 (5.4)			22–34	[8]
Pre-ART CD4 median (IQR), cells/μL	354 (248, 517)			250–550	[8]
Women virologically suppressed at delivery, %^b^	76			50–100	[8]
Time on ART prior to delivery, median (IQR), months	4 (3, 5)			0–7	[8]
B. MCH-ART intervention parameters					
	*SOC*	*MCH-ART*			
Postpartum women retained in HIV care at 1 year, %	71	81		71-91^c^	[8]
Postpartum women retained in care and virologically suppressed at 1 year, % ^b^	49^d^	67^d^		49–75 ^c^	[8]
Breastfeeding practices					
Breastfeeding duration, mean (SD), months	6 (6)	8 (6)		0–18	[8]
Exclusive breastfeeding, %^e^	71	77		0–100	[8]
Probability of infant having a 6–10 week EID test, %	78	82		0–100	[8]
Postpartum healthcare costs, 2016 USD	50	69		35–138	[8]
C. Peri- and postnatal HIV transmission risks					
Intrauterine (IU)/ intrapartum (IP) transmission, %					
On ART–virologically suppressed^b^	0.44			0.00–0.88	[13–18]
On ART–not virologically suppressed^b^	2.57			0.00–10.00	[13–18]
Postnatal transmission (during breastfeeding), %/mo.					
On ART–any breastfeeding, virologically suppressed^b^	0.05			0.00–0.10	[15, 18–20]
On ART–any breastfeeding, not virologically suppressed^b^	0.21			0.00–1.20	[15, 18–22]
C. Peri- and postnatal HIV transmission risks (cont.)					
Not on ART–exclusive breastfeeding, range by CD4	0.24–0.76			0.06–1.52	[23–30]
Not on ART–mixed/complementary breastfeeding, range by CD4	0.40–1.28			0.10–2.56	[23–30]
D. Costs (in 2016 USD)					
	*SOC*		*MCH-ART*		
Cumulative postpartum healthcare costs for first 12 months^f^	50		69	35–138	[8]
Maternal ART (per month)					
1^st^ line (TDF/FTC/EFV)	9			5–18	[31]
2^nd^ line (AZT/3TC/LPV/r)	27			14–54	[31]
Pediatric ART (range by age and weight, per month)					
1^st^ line (ABC/3TC/LPV/r)	21–44			0.5-2x	[31]
2^nd^ line (AZT/3TC/EFV)	10–25			0.5-2x	[31]
HIV routine care costs (range by CD4, per month)	17–129			0.5-2x	[32]

SD: standard deviation; IQR: interquartile range; ART: antiretroviral therapy; SOC: standard of care; EID: early infant diagnosis; USD: United States dollars; TDF: tenofovir disoproxil fumarate; FTC: emtricitabine; EFV: efavirenz; AZT: azidothymidine; 3TC: lamivudine; LPV: lopinavir; r: ritonavir; ABC: abacavir.

^a^ See S2 Table for details of literature reviews performed to inform ranges examined.

^b^ Virologic suppression defined as HIV RNA <50 copies/mL.

^c^ Range varied in *MCH-ART* strategy and compared to the *SOC* base case.

^d^ Virologic suppression among those in care was 68% with *SOC* and 80% with *MCH-ART*.

^e^ Reflects the % of breastfeeding women who were practicing exclusive breastfeeding at three months postpartum.

^f^ This cost represents the cumulative 12-month cost of providing postpartum healthcare to the mother-infant pair, and not a recurring monthly cost.

Using the CEPAC models, we projected pediatric HIV infections, 1-year mortality, undiscounted life expectancies, and undiscounted per-person HIV-related costs for women and children from a healthcare system perspective. We discounted costs and life expectancies at 3%/year to calculate an incremental cost-effectiveness ratio (ICER): the difference in lifetime HIV-related costs divided by the difference in life expectancy between the two strategies [33]. We defined a strategy as “cost-effective” if the ICER was <$903/YLS, the CEPAC-projected ICER of adding second-line ART, compared to first-line ART alone (S3 Table). Second-line ART is recommended by national guidelines in South Africa, so therefore provides a reasonable lower bound for willingness to pay [34, 35]. ICERs were reported to the nearest $1, and we performed extensive sensitivity analyses around key model parameters to evaluate the uncertainty around these estimates.

This study was approved by the Partners Healthcare Human Subjects Committee (2016P000492), Boston, MA, USA, the Institutional Review Board of Columbia University Medical Center, New York, NY, USA, and the University of Cape Town Faculty of Health Sciences Human Research Ethics Committee, Cape Town, South Africa. MCH-ART trial participants provided written informed consent.

### MCH-ART trial, modeled strategies, and population

MCH-ART (ClinicalTrials.gov NCT01933477) was a randomized controlled trial conducted in a subdistrict of Cape Town, South Africa (2013–2016) that evaluated two approaches to postpartum care for women initiating ART antenatally and their breastfed children [8]. The trial enrolled women at least 18 years of age who were less than 6 weeks postpartum (median [IQR] days postpartum: 5 [4,8]) and who had started ART during their recently completed pregnancy. In order to be eligible for trial enrollment, women had to be breastfeeding their infants at the time of screening. Mother-infant pairs enrolled in the trial were randomized to one of two arms. Standard of care consisted of immediate postnatal referral to separate general ART clinics for women and routine pediatric care for children. The MCH-ART intervention continued comprehensive maternal-pediatric care, integrated into MCH services through the end of breastfeeding. Then, women and children were referred to local clinics, as in the standard of care. A detailed costing study using bottom-up methodology was performed alongside the MCH-ART trial (S1 Appendix).

In this modeling analysis, we simulated cohorts of women and children similar to MCH-ART participants [8]. We examined two strategies that mirrored the two MCH-ART trial arms: *SOC* and *MCH-ART* [8]. ART use, testing for early infant diagnosis (EID), and laboratory monitoring were simulated in accordance with South African 2015 national guidelines [35]. We populated the CEPAC models with MCH-ART trial data on maternal retention, virologic suppression, breastfeeding practices, and EID uptake at 6–10 weeks and 18 months (S1 Table). Modeled infants in both strategies also had the opportunity for birth EID testing, with equivalent uptake in both strategies. Some children in the MCH-ART trial did not undergo EID testing, so we modeled vertical transmission rates based on published data rather than calibrating to those observed in the trial [8].

### Model structure

The CEPAC-International and CEPAC-Pediatric models are Monte Carlo state-transition models of HIV infection, diagnosis, and therapy. Their structures have previously been published and are detailed in the S1 Appendix [9–11]. Simulated women enter the CEPAC-International model with a specified distribution of age, CD4 count, and HIV RNA level. At the beginning of the simulation, women enroll in HIV care and initiate first-line ART. Individuals stop ART while lost to follow-up and resume if they return to care, either after an OD or through re-engagement. The probability of suppressing HIV RNA to <50 copies/mL by six months and the monthly risk of subsequent virologic failure are modeled relative to ART adherence; better adherence results in improved virologic outcomes.

Children are simulated in the CEPAC-Pediatrics model until age 13 and then transition to the CEPAC-International model [9, 11]. Peri- and postnatal transmission risks are based on maternal CD4 count, ART use, virologic suppression, and breastfeeding practices. Model users specify the timing and frequency of EID testing [11, 35]. Children may also receive an HIV diagnosis after presentation to care with an OD. HIV-infected children start ART immediately upon diagnosis and linkage to care.

### Model calibration and validation

We calibrated and validated the CEPAC models to maternal retention and virologic suppression targets from MCH-ART trial data (1-year targets) and data from cohorts of postpartum women on lifelong ART in sub-Saharan Africa (3-year targets; S1 Appendix, S4 Table). Virologic suppression was calibrated by simulating an improvement in adherence in *MCH-ART* relative to *SOC*, and calibrating both retention and virologic suppression to match trial data at 12 months. This “adherence adjustment” expired after 12 months, reflecting the end of the intervention; we assumed no ongoing influence of the intervention on care-seeking behaviors or ART adherence beyond 12 months postpartum. Therefore, the overall proportion of women retained in care and virologically suppressed was modeled to gradually equalize between the two strategies following the expiration of the adherence adjustment. Using the validated CEPAC models, we then projected clinical and economic outcomes beyond the 12-month scope of the trial.

### Input parameters

The simulated maternal cohort reflected MCH-ART trial participants, with mean age 28.6 years, median pre-ART CD4 count 354/μL, and 76% virologic suppression at delivery (Table 1) [8]. We simulated the proportions of women retained in care (*SOC*: 71%, *MCH-ART*: 81%) and women retained and virologically suppressed (*SOC*: 49%, *MCH-ART*: 67%) at 12 months based on MCH-ART trial data [8]. Monthly probabilities of virologic failure and LTFU after 12 months were derived from published studies (S1 Table). Data from the MCH-ART trial also informed the proportion of breastfeeding women who were virologically suppressed during each month postpartum, mean breastfeeding duration (*SOC*: 6 months, *MCH-ART*: 8 months), exclusive breastfeeding rates (*SOC*: 71%, *MCH-ART*: 77%), and 6–10 week EID uptake (*SOC*: 78%, *MCH-ART*: 82%) [8]. The probability of an infant undergoing EID testing at 18 months was assumed to be the same as the 12-month maternal retention in care that was observed in the MCH-ART trial (*SOC*: 71%, *MCH-ART*: 81%), and was varied widely in sensitivity analyses (S5 Table). We conservatively assumed no intervention-related impact on infant ART uptake, retention in care, or virologic suppression. Women who were lost to follow-up were assumed to have the same monthly probability of breastfeeding their infants as those who remained in care. For breastfeeding women lost to follow-up, we assumed no use of ART, and we applied postpartum transmission risks associated with untreated HIV. We assumed that breastfeeding did not improve infant survival, and we challenged this assumption in sensitivity analyses. Full input specifications are available in S1 Table. [13–32]

While in HIV care, simulated individuals accumulate CD4-stratified monthly routine care costs (Table 1). For the first 12 months, routine care costs (exclusive of diagnostic and drug costs) were replaced by the cumulative postpartum healthcare costs for the mother-child pair (SOC: $50, MCH-ART: $69) observed in the MCH-ART costing study (S1 Appendix) [8]. Costs were adjusted using GDP deflators and converted to 2016 US dollars [36, 37]. There was no additional adjustment for purchasing power parity, because in the absence of microcosting data, we were unable to distinguish tradeable from non-tradeable goods.

### Sensitivity analyses

We performed extensive one- and multi-way deterministic sensitivity analyses, consistent with International Society for Pharmacoeconomics and Outcomes Research-Society for Medical Decision Making (ISPOR-SMDM) guidelines [11, 38]. We investigated the impact of changes in cohort characteristics, maternal retention and virologic suppression, duration of the MCH-ART intervention, routine care costs, EID uptake, and breastfeeding practices (S1 and S2 Tables). We performed sensitivity analyses evaluating an increased risk of infant mortality with replacement feeding. We also varied *MCH-ART* postpartum healthcare costs to address concerns about the potential for longer clinic visits leading to increased staffing needs with the intervention at scale. To assess uncertainty in the durability of the effect of the MCH-ART intervention, we examined the cost-effectiveness of *MCH-ART* if the effect of the intervention waned more rapidly after the end of the intervention than in the base case. In this analysis, rates of maternal loss to follow-up and ART non-adherence between 12–24 months postpartum were modeled as higher in *MCH-ART* than in *SOC*, to simulate a situation in which the percent of women retained and virologically suppressed in this strategy equalized to *SOC* estimates by 12 months post-intervention (24 months postpartum). In multi-way analyses, we simultaneously varied combined maternal retention and virologic suppression at 12 months, breastfeeding duration, and postpartum healthcare costs.

### Budget impact analysis

We conducted a budget impact analysis to assess the short-term financial implications of adopting the MCH-ART integrated care strategy for all postpartum women with HIV in South Africa. Using the healthcare system perspective, we calculated cumulative, undiscounted costs over 2- and 5- year periods for the estimated 250,000 women eligible for the MCH-ART intervention each year and their children [1].

## Results

### Base case results

**Fig 1:**
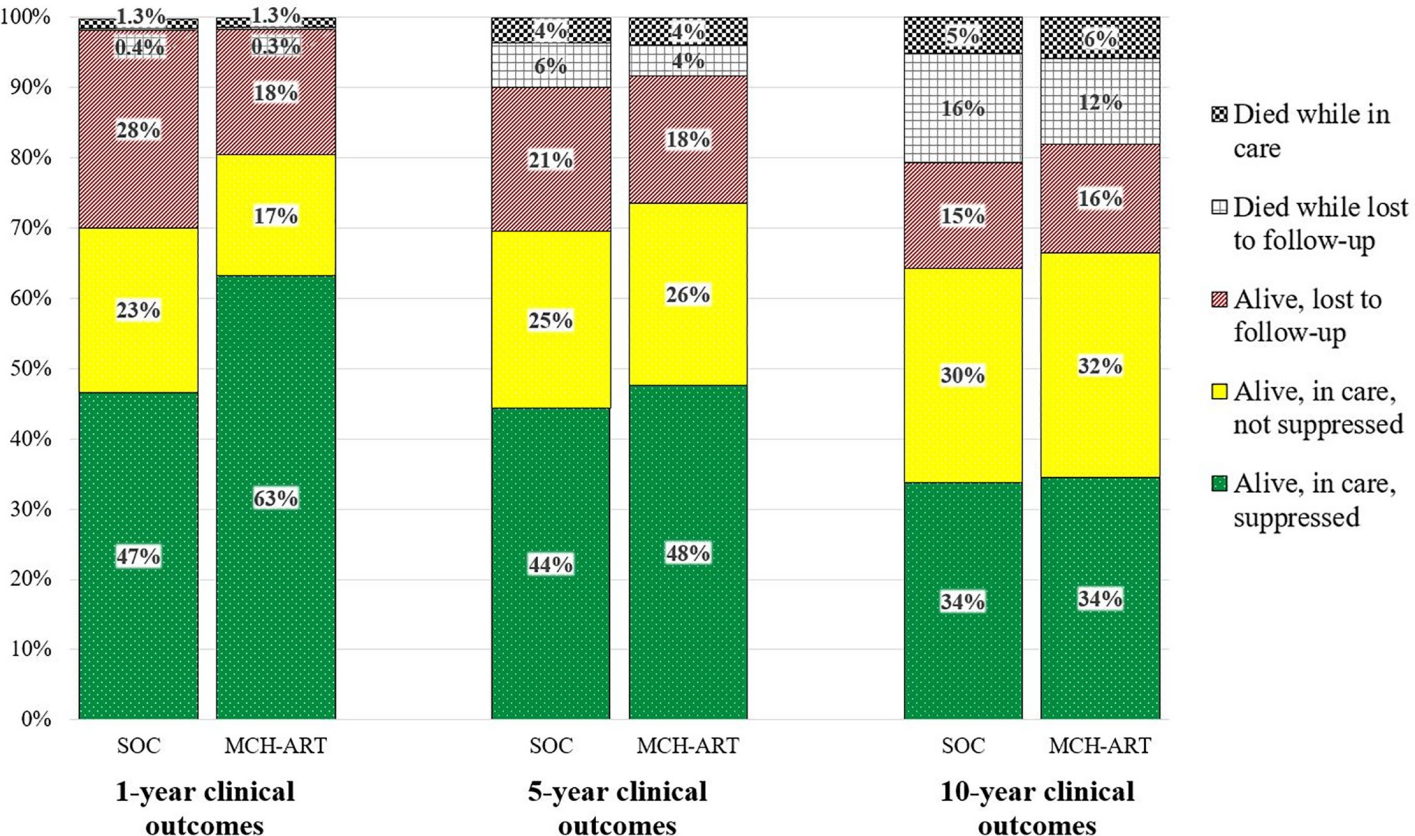
Maternal care cascade through 10 years postpartum. Projected outcomes for the starting maternal cohort at 1, 5, and 10 years postpartum are shown for the standard of care (*SOC*; left bar) and *MCH-ART* (right bar) strategies. Cumulative maternal mortality at each time point is demonstrated by the checkered black (while lost to follow-up) and gray grid (while in care) sections. Among mothers alive at 1 year, 5 years, and 10 years, those lost to follow-up (red, diagonal lines), in care and not virologically suppressed (solid yellow), and in care and virologically suppressed (green, dotted) are depicted.

Projected one-year maternal mortality was similar in both strategies (*SOC*: 1.7%; *MCH-ART* 1.6%; Fig 1 black checkered + gray grid sections), but diverged by 10 years (*SOC*: 21%; *MCH-ART*: 18%). In *SOC*, 27% of mothers were missing from care at 5 years postpartum, as 6% had died while lost to follow-up (gray grid section; 23% of missing) and 21% were alive and lost to follow-up (red diagonal section; 77% of missing). In *MCH-ART*, 22% of mothers were missing from care at 5 years postpartum, including 4% who had died while lost to follow-up (20% of missing) and 18% who were alive and lost to follow-up (80% of missing). The overall percent of women who were in care and virologically suppressed equalized at 6 years after the end of the intervention and remained equal through 10 years postpartum (green dotted section). By 10 years postpartum, among the 31% and 28% mothers missing from care in *SOC* and *MCH-ART*, 51% in SOC and 44% in MCH-ART had died while LTFU.

Maternal undiscounted life expectancy from delivery was projected to be 25.26 years in *SOC* and 26.20 years in *MCH-ART* (Table 2). Modeled rates of pediatric HIV infection were similar in both strategies (*SOC*: 2.08%, *MCH-ART*: 2.06%). For the cohort of all HIV-exposed children, life expectancies were also similar between strategies (*SOC*: 62.23 years, *MCH-ART*: 62.25 years), which resulted in similar projected HIV-free survival in both strategies. Life expectancy for HIV-infected children was slightly lower in *SOC* (23.13 years) compared to *MCH-ART* (23.40 years) due to slightly higher EID uptake, leading to faster linkage to care and HIV treatment initiation in *MCH-ART*. Overall discounted HIV-related cost per mother-child pair was $10,173 with *SOC* and $10,487 with *MCH-ART*. With discounted, combined maternal and pediatric LE and costs, *MCH-ART* was cost-effective compared to *SOC*, with an ICER of $599/YLS.

**Table 2:** Base case results.

	Maternal^a^		Pediatric^a^					Maternal + Pediatric^a^		
			HIV-infected children			All HIV-exposed children				
Strategy	LE, years (disc)	Lifetime cost, US $ (disc)	HIV infection, %^b^	LE, years	Lifetime cost, US $	LE, years (disc)	Lifetime cost, US $ (disc)	LE, years (disc)	Lifetime cost, US $^c^ (disc)	ICER, $/YLS^d^
Base case										
*SOC*	25.26 (15.98)	16,119 (9,912)	2.08	23.13	14,617	62.23 (26.09)	346 (210)	87.49 (42.07)	16,515 (10,173)	Comparator
*MCH-ART*	26.20 (16.50)	16,697 (10,207)	2.06	23.40	14,674	62.25 (26.10)	346 (211)	88.45 (42.60)	17,112 (10,487)	599

LE: life expectancy; ICER: incremental cost-effectiveness ratio; YLS: year of life saved; SOC: standard of care.

^a.^ Undiscounted life expectancy and cost projections are shown without parentheses; discounted values are shown in parentheses. All costs are reported in 2016 US$. Undiscounted maternal life expectancy was projected from delivery and pediatric life expectancy was projected from birth. Life expectancy and costs contributing to the ICER were also discounted at a rate of 3% where relevant to cost-effectiveness outcomes (shown in parentheses). ICERs were calculated from discounted values prior to rounding.

^b.^ *SOC*: Intrauterine [IU]/Intrapartum [IP] 0.95%, postpartum 1.13%; *MCH-ART*: IU/IP 0.95%, postpartum 1.11%.

^c.^ Combined maternal + pediatric costs also include undiscounted postpartum healthcare costs for the first 12 months for the mother-child pair.

^d.^ We considered an ICER <$903/YLS to be cost-effective (see Methods).

### One-way sensitivity analyses

**Fig 2:**
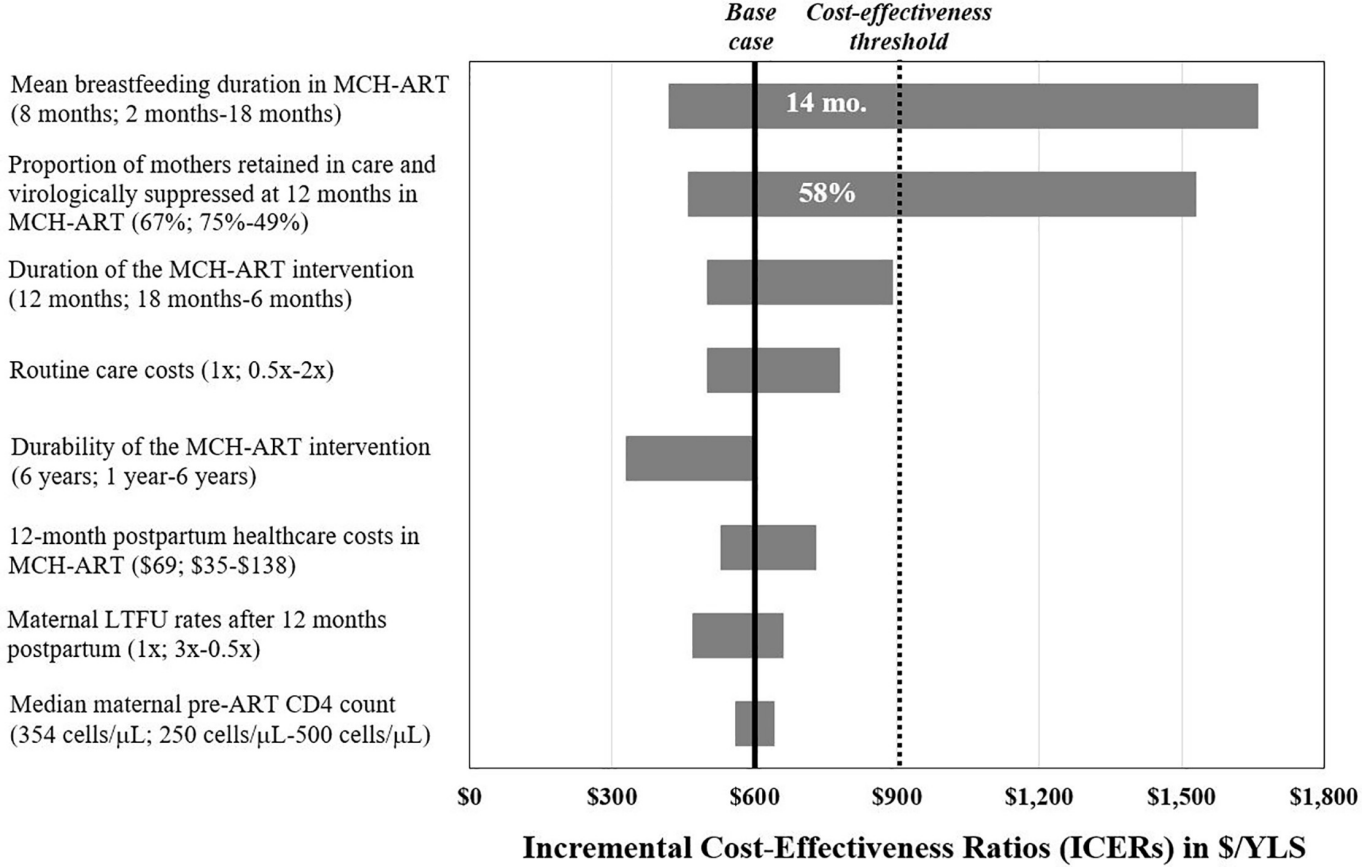
Tornado diagram of one-way sensitivity analyses. Results of one-way sensitivity analyses of 8 key model input parameters (vertical axis) are shown. The ICER ($/YLS) of *MCH-ART* compared to *SOC* is displayed along the horizontal axis. Base case inputs for each varied parameter are listed prior to the semicolon; after the semicolon is the range examined with the first value indicating the value of the left-most part of the bar. Parameters were varied through the range listed (S2 Table details rationale for ranges), with the input that contributes to the lowest ICER for the range listed first. The thick black vertical line marks the base case ICER ($599/YLS). The dotted line represents the cost-effectiveness threshold ($903/YLS; see text). Numbers in white reflect the value at which parameters crossed the cost-effectiveness threshold. *ICER*: *incremental cost-effectiveness ratio; ART*: *antiretroviral therapy; LTFU*: *loss to follow-up; YLS*: *year of life saved*.

In one-way sensitivity analyses, conclusions were sensitive to changes in two key parameters (Fig 2). First, if the mean breastfeeding duration in *MCH-ART* increased to 14 months (base case: 8 months), without changing the mean breastfeeding duration in *SOC* (base case: 6 months), *MCH-ART* was no longer cost-effective due to more pediatric HIV infections from breastfeeding. However, the longer breastfeeding duration in *MCH-ART* offered a mortality benefit in sensitivity analyses with increased relative risk of infant mortality with replacement feeding (S3 Fig). Second, *MCH-ART* was also no longer cost-effective if the proportion of mothers retained in care and virologically suppressed at 12 months in *MCH-ART* was less than 58% (base case: 67%, compared to 49% in *SOC*). *MCH-ART* remained cost-effective despite wide variations in all other input parameters, including the duration of the intervention, routine care costs, postpartum healthcare costs, maternal LTFU after 12 months, rates of long-term maternal re-engagement in care, EID uptake at all timepoints, and maternal pre-ART CD4 count (Fig 2; S5 Table). Notably, *MCH-ART* remained cost-effective even when the effect of the intervention waned completely over 1 year, resulting in equivalent proportions of women retained and virologically suppressed at 1 year post-intervention (base case: outcomes equalize at 6 years), with no lingering benefit from the intervention after 1 year.

### Multi-way sensitivity analyses

**Fig 3:**
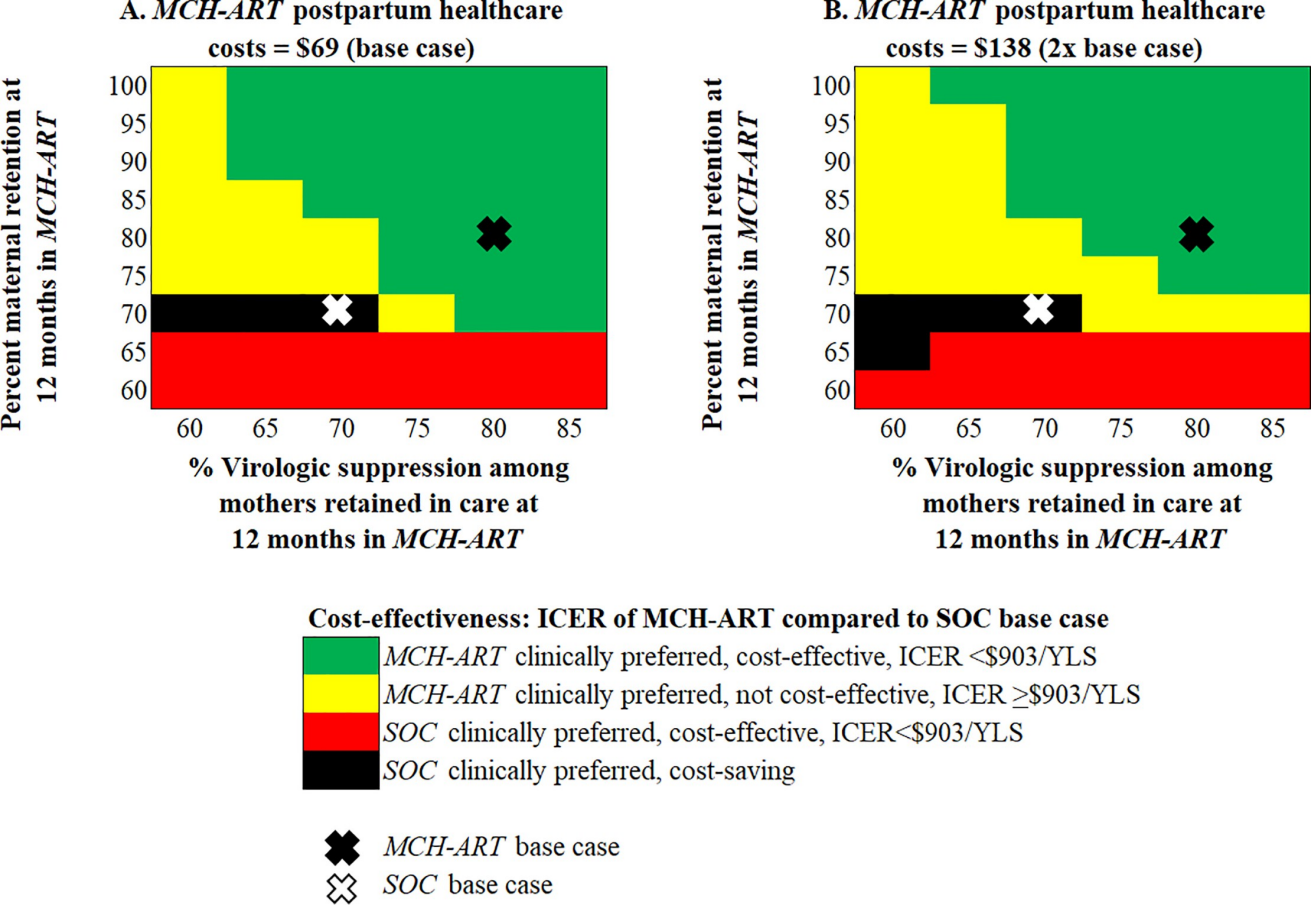
Multi-way sensitivity analyses. In panels A and B, the proportion of mothers retained in care at 12-months in *MCH-ART* is demonstrated on the vertical axis and the proportion of virologically suppressed mothers among those retained in care in *MCH-ART* is shown on the horizontal axis. Without commensurate improvements in suppression, increasing retention alone results in *MCH-ART* being no longer cost-effective (yellow). *MCH-ART* was cost-effective at a wider range of combinations of maternal retention and virologic suppression among mothers retained in care with *MCH-ART* postpartum healthcare costs at $69 (A, base case costs) compared to $138 (B, 2x base case costs). *ICER*: *incremental cost-effectiveness ratio; SOC*: *standard of care; YLS*: *year of life saved*.

In multi-way analyses, we simultaneously varied maternal retention at 12 months postpartum in *MCH-ART* (base case: *MCH-ART*: 81%, *SOC*: 71%), virologic suppression among mothers retained in care (base case: *MCH-ART*: 80%, *SOC*: 68%), and *MCH-ART* postpartum healthcare costs (base case: *MCH-ART*: $69, *SOC*: $50), holding all other parameters equal to the base case (Fig 3). Increasing retention in *MCH-ART* without increasing virologic suppression resulted in ICERs >$903/YLS. With both base case ($69, Fig 3A) and 2x base case ($138, Fig 3B) *MCH-ART* postpartum healthcare costs, no level of retention achieved in *MCH-ART* was cost-effective compared to the *SOC* base case if only 60% of mothers in care had virologic suppression. As virologic suppression among those in care improved, *MCH-ART* became increasingly cost-effective.

### Budget impact analysis

For the approximately 250,000 women with HIV in South Africa becoming pregnant each year (1.25 million women over five years), providing HIV-related care for both mothers and children led to estimated costs of $384 million ($1,032/mother-child pair) over two years and $1.94 billion ($2,603/pair) over five years. The MCH-ART intervention increased costs to $392 million ($1,071/pair) over two years and $1.99 billion ($2,683/pair) over five years. Widespread uptake of the MCH-ART platform would require a 2.0% increase in the total 2-year budget for these postpartum women with HIV and their children, and a 2.3% increase in the 5-year budget. We projected 1,710 maternal and 60 child deaths averted by the MCH-ART intervention at 2 years and 10,900 maternal and 230 child deaths averted by 5 years.

## Discussion

We evaluated integrated postpartum maternal ART and pediatric care using a detailed microsimulation model of HIV disease, populated with data from the MCH-ART trial. The MCH-ART trial demonstrated improved maternal engagement and virologic suppression with the MCH-ART intervention compared to the standard of care [8]. Other studies have demonstrated a similar, positive effect of integrated services on retention, but to our knowledge, MCH-ART is the first to show improvement in both retention and virologic suppression [39, 40]. Our results demonstrate that, in addition to being clinically effective, the MCH-ART intervention is cost-effective, with an ICER of $599/YLS. The MCH-ART approach to integrated postpartum care is projected to require a modest increase in resources over 5 years, increasing costs by $44 million (2.3%) while averting over 11,000 deaths among 1.25 million postpartum mothers with HIV and their children, and offers excellent value in South Africa given a wide range of clinical and cost assumptions.

Our findings highlight the substantial adverse impact of maternal disengagement from care on long-term maternal and pediatric health outcomes. By 5 and 10 years postpartum, approximately 25% and 50% of mothers with HIV who were lost to follow-up were projected to have died. These estimates are consistent with data from observational cohorts of adults with HIV in sub-Saharan Africa, although these studies are not specific to postpartum women who may have higher rates of re-engagement in ART care through subsequent pregnancies [41, 42]. However, fundamental conclusions about the cost-effectiveness of integrated care did not change in sensitivity analyses that explored higher rates of re-engagement in care over time. Adverse maternal outcomes are also associated with increased pediatric morbidity and mortality [43]. While increasing maternal retention in care in the first year postpartum improves maternal and pediatric clinical outcomes, our results suggest that the cost-effectiveness of a retention intervention may be compromised without corresponding improvement in virologic suppression among those retained. These findings support placing emphasis on the whole care cascade, e.g., maternal retention in care, ART adherence, and virologic suppression, when evaluating interventions aiming to improve maternal and child health.

With the breastfeeding duration observed in the MCH-ART trial, we found that pediatric outcomes are most strongly related to maternal care engagement in the first year postpartum. During this window, postnatal HIV transmission risks are highest, and EID is critical to promoting early pediatric ART initiation. Using the 6–10 week EID uptake observed in the MCH-ART trial, we found that integrated care may result in slightly better survival among HIV-infected infants due to earlier HIV detection and ART initiation. However, with the recent increase in EID testing at birth in South Africa, the small difference in 6–10 week EID uptake observed in the MCH-ART trial may no longer be clinically relevant [44]. As the burden of new pediatric infections continues to shift postpartum, maternal and infant retention in care through completion of the EID cascade, e.g. completion of pediatric HIV testing at the end of the breastfeeding period, will become increasingly important [1].

Breastfeeding is an important factor in overall infant health and survival, though its benefits can be offset by extended risks of postnatal transmission if mothers are not virologically suppressed [43]. In both arms of the MCH-ART trial, the median duration of breastfeeding was lower than the World Health Organization-recommended 12 months of breastfeeding for women with HIV in resource-limited settings who are supported on ART [45]. With this relatively short window of risk for HIV transmission and high proportion of women who were virologically suppressed in the early postpartum period, we projected low overall postnatal transmission with both *MCH-ART* and *SOC*, similar to the risks reported for other cohorts of women taking three-drug ART during breastfeeding [15, 46, 47]. Under the base case assumption that breastfeeding offered no health benefit to infants, the longer window of potential postnatal transmission in MCH-ART was almost exactly offset by lower transmission risks from improved maternal virologic suppression. Thus, projected HIV-free child survival in both strategies was similar. However, in many resource-limited settings, particularly settings where water supplies are unsafe or quantities of formula are nutritionally inadequate, breastfeeding reduces risks for bacterial infections, malnutrition, and mortality relative to replacement feeding [43]. Services that improve postpartum engagement in care while also supporting safe breastfeeding practices in these settings are critical to promoting healthy, HIV-free child survival.

As there is no consensus on the best cost-effectiveness threshold to use in economic evaluations in resource-limited settings, we used the CEPAC models to determine a cost-effectiveness threshold based on the benchmark intervention of second-line ART in South Africa [34, 48–50]. In the absence of robust pediatric health utility data from sub-Saharan Africa, we reported results in life years saved rather than quality-adjusted life years (QALYs) saved or disability-adjusted life-years (DALYs) averted. However, conclusions about the cost-effectiveness of integrated postpartum care are unlikely to change with use of health-related quality of life metrics, as quality of life would not be expected to differ substantially between strategies for this population of women healthy enough to become pregnant [51]. Our cost-effectiveness threshold of $903/YLS is similar to the threshold range identified in the South African HIV Investment Case (ICER: $547-$872/YLS), which explored the optimal combination of HIV-related interventions to improve health outcomes given budgetary constraints in South Africa [50]. The ICER of *MCH-ART* versus *SOC* (ICER: $599/YLS) compares favorably to that of other HIV-related MCH interventions in South Africa, including a rapid ART initiation program in pregnancy (ICER: $1,160/quality-adjusted life year saved) and infant birth EID testing (ICER: $2,206-$2,900/YLS) [50, 52, 53]. Published ICERs of infant birth EID testing, which is widely implemented in South Africa, are also higher than our cost-effectiveness threshold of $903/YLS, which suggests that this threshold is conservative; true willingness to pay for the health of HIV-exposed infants may be higher in South Africa. Our findings suggest that, regardless of the approach used to determine the cost-effectiveness threshold, integrated postpartum care is likely to offer good value relative to other health interventions in South Africa.

Prior modeling analyses have demonstrated the cost-effectiveness of integrating HIV care with family planning services, cervical cancer screening, and tuberculosis screening [6, 54]. Integration of these services may increase the efficiency of service delivery while also improving patient satisfaction [6]. However, concerns have been raised that service integration could increase workload among clinic personnel, leading to accelerated provider burnout, reduced quality of care, and prolonged patient waiting times, any of which could undermine clinical- and cost-effectiveness at scale [6, 7]. We performed a multi-way sensitivity analysis of postpartum healthcare costs in *MCH-ART* and maternal retention and virologic suppression to assess the potential impact of increased costs related to longer patient visit times and/or increased personnel needs with larger patient volumes at scale. With the level of maternal engagement in care observed in the MCH-ART trial, the integrated care intervention remained cost-effective even with postpartum healthcare costs 2-fold higher than measured in the MCH-ART costing study [8].

The durability of impact from integrated care interventions like MCH-ART in the long run remains unclear. We conservatively assumed no improvement in care engagement from the MCH-ART intervention after 12 months postpartum, which resulted in the gradual equalization of outcomes by 6 years after the end of the intervention. If the intervention proves more lasting in promoting continued care engagement, then we may have underestimated its value. Conversely, the MCH-ART intervention may only delay “inevitable” post-breastfeeding attrition after the intervention ends. We evaluated this possibility in our sensitivity analysis of the durability of the intervention, which showed that *MCH-ART* remained cost-effective, even if any improvement in overall engagement in care had completely faded by 1 year after the end of the intervention. Over a lifetime time horizon, the absolute projected clinical benefit from the 12-month MCH-ART intervention was small, but the intervention still offered good value at relatively low cost. While innovative integrated service delivery platforms are implemented and scaled up in resource-limited settings, further research is needed to assess tradeoffs in costs, provider workload, patient satisfaction, and quality of care, as well as the durability of impact on patient outcomes over time.

Our analysis has several limitations. First, as with any model-based analysis, uncertainty is inherent in long-term projections. Few data exist to inform patterns of long-term retention and ART adherence among women who initiate ART during pregnancy, and retention is variably defined [55]. However, we calibrated our model to the best available data for up to three years postpartum. Longitudinal observational studies are needed to confirm the long-term clinical outcomes and cost-effectiveness of integrated postpartum care in a “real-world” context. Second, we did not account for subsequent pregnancies that may attenuate budget impact or prompt re-engagement in care. Keeping women engaged and virologically suppressed on ART as they enter a second pregnancy may have important impacts on maternal health, obstetric outcomes, and perinatal HIV transmission, and could improve the value of the intervention [56]. Third, to reflect the MCH-ART trial, we simulated only breastfeeding women who were not already taking ART at the beginning of pregnancy. Women whose infants are formula-fed or who are on ART at conception (approximately 37% of the population screened for the MCH-ART trial), may have retention and adherence behaviors that would be affected differently by integrated services [8]. However, a recent study from South Africa found that women who conceived while on ART had higher rates of postpartum virologic failure than those who started ART during pregnancy, and thus may also benefit from interventions to improve care engagement and ART adherence [57]. Finally, our model input data and assumptions are informed by the South African healthcare system. Nonetheless, model outcomes may still be generalizable outside of this context, as the results were robust to wide variations in maternal characteristics, timing of antenatal ART initiation, EID uptake, and routine care costs, all relevant to a range of other resource-limited settings.

## Conclusions

The MCH-ART trial showed that integrating postpartum maternal ART and pediatric care into MCH services improves clinical outcomes at one year. We found that this intervention is likely to lead to improved life expectancy among mothers and HIV-infected infants and is cost-effective in South Africa. This approach to integrated postpartum care should be more widely implemented to improve maternal and child health.

## Supporting information

S1 Checklist Consolidated health economic evaluation reporting standards (CHEERS) checklist. (DOCX)

S1 Appendix Provides further detail regarding modeling methods, CEPAC structure, model calibration, and methods for sensitivity and scenario analyses. (DOCX)

S1 Fig Cohort size estimation. The discounted ICER (blue line) converged at cohort sizes (horizontal axis) of 10 million and greater for base case maternal and pediatric simulations for *SOC* and *MCH-ART*. Therefore, we chose a cohort size of 10 million for all simulations to produce stable per-person estimates.
(TIFF)

S2 Fig One-way sensitivity analysis of pediatric HIV infection and breastfeeding duration. Projected pediatric HIV infection rates for the *SOC* and *MCH-ART* base case duration of breastfeeding (blue and red dots) and 12-month duration of breastfeeding (blue and red squares) are shown.
(TIFF)

S3 Fig One-way sensitivity analysis of five-year HIV-free child survival with increased relative risk of non-AIDS mortality with replacement feeding (RR-RF). Base case (RR-RF = 1) assumes no increased relative risk of mortality with replacement feeding as compared to breastfeeding.
(TIFF)

S1 Table Input parameters for a simulation model of women with HIV and their children in South Africa. (DOCX)

S2 Table Sources of data and ranges for sensitivity analyses. (DOCX)

S3 Table Model output used to determine the cost-effectiveness threshold. (DOCX)

S4 Table Model calibration and validation of maternal engagement in care. (DOCX)

S5 Table Results of selected one-way sensitivity analyses. (DOCX)
