# Supplementary material for: Cost-effectiveness of integrating postpartum antiretroviral therapy and infant care into maternal & child health services in South Africa
Source: PLoS One. 2019 Nov 15;14(11):e0225104. doi: 10.1371/journal.pone.0225104 (PMC6857940; doi:10.1371/journal.pone.0225104)
Supplement: S1 Appendix — (DOCX) [file pone.0225104.s002.docx]

**Cost-effectiveness of integrating postpartum antiretroviral therapy and infant care into**

**maternal & child health services in South Africa**

**Technical Appendix**

**Dugdale et al.**

This technical appendix supplements the manuscript by providing additional details regarding modeling methodology, input specifications, and derivation of key parameters. Further information about the Cost-effectiveness of Prevention AIDS Complications (CEPAC)-International and CEPAC–Pediatric models is available in previously published works [1-11]. For more details regarding the mathematical formulas used in the model, model flowcharts, examples of source code, and opportunities for collaboration, we direct readers to the CEPAC website: <http://www.massgeneral.org/mpec/cepac/>.

**Methods**

*Cohort size and characteristics*

Using the CEPAC-International model, we simulated a cohort of pregnant women with HIV, who had initiated antiretroviral therapy (ART) while attending antenatal care, from delivery through death. We simulated a second cohort of HIV-exposed infants born to these women using the CEPAC-Pediatric model. Both HIV-infected and HIV-uninfected children were simulated over the course of their lifetimes. In order to reduce stochastic uncertainty, we evaluated the optimal cohort size for these simulations (Figure S1) [12]. Model output remained stable at run sizes of 10 million per cohort and greater, so we used a cohort size of 10 million to generate all output for this analysis. We then applied per person outcomes to setting-specific population sizes (e.g. the number of women with HIV in South Africa who become pregnant each year).

*Model structure and input parameters*

Antepartum initialization. In the MCH-ART trial, women were screened for eligibility, had baseline CD4 and HIV viral load measurements, and were initiated on 3-drug ART with tenofovir/emtricitabine/ efavirenz (TDF/FTC/EFV) during the antepartum period [13]. ART was started at a median of 4 months (IQR 3-6) prior to delivery [13]. To capture the impact of antepartum ART initiation, we performed an initialization run using MCH-ART observational cohort characteristics and adjusted the starting CD4 count at delivery in the base case runs to that predicted by a simulation of four months of ART use [13]. CD4 counts are lower in pregnancy due to volume of distribution and immunologic factors that are not associated with an increased risk of opportunistic diseases (ODs) or other HIV-related outcomes [14]. Therefore, we also mapped the starting antepartum CD4 distributions of women enrolled in the trial to expected physiologic postpartum CD4 values as part of this initialization run [14].

Adherence and ART efficacy. All simulated women with HIV start first-line ART at the beginning of the simulation with a 95% probability of virologic suppression based on data from the MCH-ART trial [13]. While virologically suppressed, simulated patients experience a monthly rise in their CD4 count [15, 16]. Use of ART also results in attenuated risks of ODs and AIDS-related mortality, independent of virologic suppression [1, 17].

To simulate the heterogeneity of individual patients’ responses to ART in the CEPAC models, all patients are assigned a “propensity to respond” (PTR) coefficient that specifies their ART adherence and care-seeking behavior throughout the simulation. PTR coefficients are drawn from a normal distribution with a user-defined mean and standard deviation. This distribution is then logit-transformed to yield a range of values between 0 and 1, with each value representing the % adherence. Probabilities of initial virologic suppression (HIV RNA <50 copies/mL) within six months on ART, virologic failure after initial suppression, and loss to follow-up are specified for the highest and lowest adherence buckets and exponentially interpolated in between [18]. Individuals with better adherence (e.g. higher PTR coefficient) are more likely to achieve and maintain virologic suppression on ART than those with poorer adherence (e.g. lower PTR coefficient; Table S3). Patients with better adherence are also less likely to become lost to follow-up. This modeling approach has previously been described [18].

For this analysis, we expanded upon the existing CEPAC PTR coefficient framework to develop a novel approach to modeling adherence interventions in a heterogeneous population. Upon initiation of the simulation, individuals can receive an “adherence adjustment,” which applies a time-limited PTR-adjustment to reflect a temporary change in their care-seeking behaviors (e.g. retention in care and ART adherence). To replicate the increase in engagement with the MCH-ART intervention observed in the MCH-ART Trial, we applied a positive PTR coefficient adjustment in the modeled *MCH-ART* *strategy* for a mean of 12 (SD = 3) months. In the *SOC* *strategy*, individuals did not receive a PTR coefficient adjustment. The PTR coefficient, PTR coefficient adjustment, and LTFU probabilities in each modeled strategy were calibrated to replicate the retention in care and virologic suppression rates at 12 months postpartum in the two arms of the MCH-ART Trial (Table S2). After 12 months, the PTR-coefficient adjustment expired, representing the end of the MCH-ART intervention.

Loss to follow-up and return to care. When simulated patients become lost to follow-up (LTFU), they stop attending clinic visits and discontinue ART and co-trimoxazole prophylaxis. If ART is discontinued, the HIV viral load increases back to the starting setpoint, and the CD4 count declines rapidly [19, 20]. After hitting the viral load setpoint, simulated patients experience HIV natural history rates of CD4 decline, ODs, and AIDS-related death (Table S3). If adults who are LTFU experience a WHO Stage III or IV OD, they have a 50% probability of returning to care, whereas children were given a 100% probability of returning to care. Patients who are LTFU also have a “background” probability of returning to care, independent from OD-driven return to care, after a minimum of 6 months out of care [21, 22].

When individuals return to care, they reinitiate ART. Patients who were previously suppressed on ART return to the same line of ART they were taking prior to being LTFU, with a 96% chance of resuppression on that line [23]. If they were failing ART prior to being LTFU, but not yet recognized as failing, they return to the line of ART they were taking previously. If they were recognized as failing ART at the time of becoming lost, but had not yet transitioned to the next ART line, they initiate the next ART line when returning to care. Patients with poorer adherence (e.g. lower PTR coefficients) have a higher probability of becoming LTFU (Table S3), consistent with studies that have shown an increased risk of disengagement in care among those who are non-adherent to ART while in care [24, 25].

Peri- and postnatal HIV transmission. All HIV-exposed infants experience a one-time risk of intrauterine(IU)/intrapartum(IP) HIV infection, followed by monthly risks of postnatal HIV infection while breastfeeding. Postnatal transmission risks were calculated under the assumption that infants received six weeks of appropriate antiretroviral prophylaxis if their mothers were on ART [26]. We stratified peri- and postnatal transmission risks by maternal CD4 count, ART use, and virologic suppression. We derived one-time IU/IP transmission risks based on virologic suppression at delivery from published studies of mothers who were started on three-drug ART during the index pregnancy [27-32]. We derived postnatal transmission risks from published literature that reported virologic suppression in association with overall pediatric HIV infection rates, maternal ART use, and breastfeeding duration [29, 31, 33-36]. Transmission rates for mothers who were not on ART were derived from studies in the pre-Option B+ era and were stratified by maternal CD4 count and breastfeeding practice [37-44]. Rates of maternal ART use and virologic suppression varied by month throughout breastfeeding and were derived from MCH-ART Trial data. We assume that women who are lost to follow-up prior to 12 months postpartum continue breastfeeding for the same duration as women who are still in follow-up in the standard of care arm of the trial.

Early infant diagnosis. All children encounter opportunities for early infant diagnosis (EID) testing in the CEPAC-Pediatric model. The probabilities of presenting for an EID test at birth and 6-10 weeks were informed by both MCH-ART Trial data and published literature [13, 45]. Final rates of 18-month EID test uptake were not available directly from the MCH-ART Trial. Therefore, we assumed that differences in 18-month EID uptake mirrored differences in maternal retention in care at 12 months in the MCH-ART Trial and varied this parameter widely in sensitivity analyses (Table S3) [13]. In the CEPAC-Pediatric model, infants experience delays in result return consistent with laboratory-based testing programs [46]. Those who test positive undergo confirmatory testing with a second nucleic acid test [11]. For HIV-positive infants who miss testing opportunities prior to 18 months or who do not become infected until after the last scheduled EID test, diagnosis and linkage to care can only be achieved following an OD.

HIV-related care costs. The costs of WHO stage 3 or 4 ODs and tuberculosis for patients under 5 years old were derived from a previously published study of healthcare costs for HIV-infected children from South Africa [47, 48]. Costs of OD-related care for individuals more than 5 years old were calculated by multiplying resource use (e.g. outpatient visits, inpatient days, laboratory testing, and medication use) from the Cape Town AIDS Cohort by South African unit costs [47, 49]. Adult ART costs for first-line TDF/FTC/EFV and second-line zidovudine, lamivudine, and boosted lopinavir (AZT/3TC/LPV/r) were taken directly from the Clinton Health Access Initiative (CHAI) price list [50]. Pediatric ART costs were also taken from CHAI, but then adjusted for weight, age, and ARV formulation with monthly costs that ranged from $10-$44 [50, 51].

While in HIV care, simulated individuals accumulate CD4-stratified routine care costs reflecting the costs of clinic infrastructure, provider time, and laboratory monitoring other than CD4 and HIV viral load tests, ranging from $17 to $129 per month [47, 49]. During periods of LTFU, we assume that individuals receive 20% of monthly routine care costs, and we varied this parameter widely in sensitivity analyses.

For the first year of the simulation, we extracted all model-based routine care costs and replaced these values with the 12-month cumulative postpartum healthcare costs calculated in a detailed costing study that was performed alongside the MCH-ART trial [13]. This costing analysis accounted for infrastructure costs, overheads, provider time, and staff salaries, both for the MCH-ART intervention setting of the Midwife Obstetric Unit and for standard of care clinic service delivery [13]. The personnel and infrastructure costs were calculated using bottom-up methodology including the use of staff time-sheets, space measurements, furniture and equipment lists, while overhead costs followed top-down methodology of allocation of costs based on utilization rates. Costs per clinic visit were allocated to the standard of care (*SOC*) and MCH-ART intervention (*MCH-ART*) strategies based on postpartum clinic visit attendance in local clinics and in the Midwife Obstetric Unit, which houses Maternal and Child Health services. Using clinic visit attendance data from the MCH-ART Trial, 12-month cumulative postpartum healthcare costs, excluding the cost of ART and laboratory monitoring, were $50 in *SOC* and $69 in *MCH-ART*.

*Model calibration*

The relationship between ART adherence and LTFU was informed by data from a study of postpartum retention in care among women on lifelong ART through Malawi’s Option B+ program [24]. In Malawi, women with <85% cumulative medication adherence at six months had a 1.94-fold greater risk of becoming LTFU than those with >95% adherence [24]. Keeping the ratio of the monthly probabilities of LTFU with adherence <85% and >95% at 1.94, we calibrated the PTR coefficient adjustment and monthly risks of LTFU in the first year to reach total proportions of women alive and retained in HIV care at 12 months of 71% in *SOC* and 81% in *MCH-ART* to match MCH-ART Trial data (Table S3) [13]. The PTR coefficient, PTR coefficient adjustment, and monthly probabilities of virologic failure were also calibrated to closely match 12-month virologic suppression targets of 49% and 67% in the *SOC* and *MCH-ART* strategies [13]. After 12 months, the PTR coefficient adjustment expired, representing the end of the intervention. Adherence-stratified monthly risks of LTFU and risks of virologic failure were then equal between strategies and were calibrated to match 3-year calibration targets in *SOC* for retention and suppression from the published literature (Table S2) [24, 52-54]. We also performed calibration to match maternal postpartum CD4 trajectories while LTFU to the CD4 trajectories of postpartum women who stopped ART as part of the HAART standard component of the Promoting Maternal and Infant Safety Everywhere (PROMISE) trial [19].

*Model validation*

Prior published CEPAC studies have reported details of calibration and validation of projected ODs and survival for South African adults and HIV-infected infants [1, 2, 5]. As in prior CEPAC analyses, we assessed face validity and internal validity through the examination of model-generated traces of individual-level outcomes [5, 55]. For this analysis, we performed partially dependent external validation of maternal retention and adherence outcomes by comparing model output to data from the MCH-ART trial for 12-month outcomes and published data from Malawi, Uganda, and South Africa reporting on 2-4 year follow up for 36-month outcomes [24, 52-55]. We validated infant HIV-free survival against cross-sectional survey data collected between 2012-2013 from the South Africa PMTCT Evaluation (SAPMTCTE) study [56-59].

*Sensitivity analysis of the relative risk of non-AIDS mortality with replacement feeding*

We conducted a sensitivity analysis to evaluate the potential benefit of breastfeeding to overall infant health and survival. In our base case analysis, we conservatively assumed no benefit from breastfeeding on infant morbidity or mortality. However, a recent individual pooled analysis of studies involving HIV-exposed, uninfected (HEU) children in African and Asian settings found that HEU children who never breastfed had higher all-cause mortality at 24-months than children who were breastfed (aHR 2.5, 95% CI: 2.0-3.2) [60]. A secondary analysis of the Kesho Bora trial also demonstrated that for children born to HIV-infected pregnant women taking ART, breastfeeding for less than 3 months was associated with lower HIV-free survival than >3 months of breastfeeding (aHR 0.36, 95% CI: 0.15-0.83) [61]. To capture this increased risk of replacement feeding (RR-RF) relative to breastfeeding, as in previous work, we applied a RR-RF multiplier to the monthly non-AIDS-related mortality rate for three months after weaning in sensitivity analyses that ranged the relative risk from 1 (RR-RF =1; base case) to 3 (RR-RF = 3) with results depicted in Figure S3 [62].

*Cost-effectiveness thresholds*

While the WHO-CHOICE 1x *per-capita* GDP-based cost-effectiveness threshold (CET) has been widely cited, there is growing concern that spending to this threshold may not offer good value, particularly in resource-limited settings [63-66]. Due to budget constraints, investing in interventions at this GDP-based CET may result in substantial opportunity costs (e.g. forgone health benefits) relative to other investments in health care that offer better value [63-65]. Alternative approaches to defining the CET include comparison to “benchmark interventions,” (e.g. interventions that already have investment in the country of interest), or an “opportunity cost approach” in which interventions are compared through extensive league tables and serially evaluated until the budget is fully exhausted [66].

We used the CEPAC models to determine an appropriate cost-effectiveness threshold, based on the benchmark intervention of second-line ART, in the South African context. Using the same cohort characteristics, clinical care details, and peri- and postnatal transmission risks as specified in the SOC strategy, we compared a strategy of widely-available second-line ART to a strategy of first-line ART alone. Cost inputs for these strategies resembled the base case *SOC* *strategy*. We calculated an ICER from combined maternal and pediatric outcomes and reported it in $/YLS (Table S1). The CEPAC-generated ICER of second-line ART compared to first-line ART only was $903/YLS. The $903/YLS CEPAC-derived ICER is near the alternative CET identified by the South African Investment Case ($547-$872/YLS), which was an opportunity cost and league table-based exploration of a setting-specific CET, although direct cross-comparison is limited by differences in model structure and assumptions [65].

**Results**

*Model calibration and validation*

Model-generated maternal retention and virologic suppression output successfully approximated external validation data at 1- and 3-years (Table S2). In the validation of HIV-exposed infant outcomes, CEPAC-Pediatric output for HIV-infection and/or infant mortality was consistent with SAPMTCTE data. The projected overall rates of pediatric HIV infection for both the *SOC* and *MCH-ART* strategies through the first 12 months postpartum closely resembled the rates observed among tested infants in the MCH-ART Trial (SOC: 0.41%, MCH-ART: 1.69%) [13].

*Sensitivity analysis*

In the increased RR-RF sensitivity analysis, pediatric HIV-free survival at 5-years decreased with increasing relative risk of non-AIDS related mortality with replacement feeding. While HIV-free survival at 5-years was identical in both strategies in the base case (RR-RF = 1), the *MCH-ART strategy* improved HIV-free child survival relative to *SOC* if RR-RF = 2 or RR-RF = 3 (Figure S3). As the RR-RF increased, *MCH-ART* also became increasingly cost-effective, with ICERs of $531/YLS (RR-RF = 2) and $481/YLS (RR-RF = 3; Table S5).

**REFERENCES:**

1. Ciaranello AL, Morris BL, Walensky RP, Weinstein MC, Ayaya S, Doherty K, et al. Validation and calibration of a computer simulation model of pediatric HIV infection. PLoS One. 2013;8(12):e83389.

2. Ciaranello AL, Doherty K, Penazzato M, Lindsey JC, Harrison L, Kelly K, et al. Cost-effectiveness of first-line antiretroviral therapy for HIV-infected African children less than 3 years of age. AIDS. 2015;29(10):1247-59.

3. Francke JA, Penazzato M, Hou T, Abrams EJ, MacLean RL, Myer L, et al. Clinical impact and cost-effectiveness of diagnosing HIV infection during early infancy in South Africa: Test timing and frequency. J Infect Dis. 2016;214(9):1319-28.

4. Walensky RP, Borre ED, Bekker LG, Resch SC, Hyle EP, Wood R, et al. The anticipated clinical and economic effects of 90-90-90 in South Africa. Ann Intern Med. 2016;165(5):325-33.

5. Walensky RP, Wood R, Ciaranello AL, Paltiel AD, Lorenzana SB, Anglaret X, et al. Scaling up the 2010 World Health Organization HIV Treatment Guidelines in resource-limited settings: a model-based analysis. PLoS Med. 2010;7(12):e1000382.

6. Ciaranello AL, Perez F, Keatinge J, Park JE, Engelsmann B, Maruva M, et al. What will it take to eliminate pediatric HIV? Reaching WHO target rates of mother-to-child HIV transmission in Zimbabwe: a model-based analysis. PLoS Med. 2012;9(1):e1001156.

7. Goldie SJ, Yazdanpanah Y, Losina E, Weinstein MC, Anglaret X, Walensky RP, et al. Cost-effectiveness of HIV treatment in resource-poor settings--the case of Côte d'Ivoire. N Engl J Med. 2006;355(11):1141-53.

8. Ciaranello AL, Lockman S, Freedberg KA, Hughes M, Chu J, Currier J, et al. First-line antiretroviral therapy after single-dose nevirapine exposure in South Africa: a cost-effectiveness analysis of the OCTANE trial. AIDS. 2011;25(4):479-92.

9. Losina E, Toure H, Uhler LM, Anglaret X, Paltiel AD, Balestre E, et al. Cost-effectiveness of preventing loss to follow-up in HIV treatment programs: a Côte d'Ivoire appraisal. PLoS Med. 2009;6(10):e1000173.

10. Walensky RP, Ross EL, Kumarasamy N, Wood R, Noubary F, Paltiel AD, et al. Cost-effectiveness of HIV treatment as prevention in serodiscordant couples. N Engl J Med. 2013;369(18):1715-25.

11. Dunning L, Francke JA, Mallampati D, MacLean RL, Penazzato M, Hou T, et al. The value of confirmatory testing in early infant HIV diagnosis programmes in South Africa: A cost-effectiveness analysis. PLoS medicine. 2017;14(11):e1002446.

12. Law AM. Simulation modeling and analysis. 5th ed. New York: McGraw-Hill; 2015. 776 p.

13. Myer L, Phillips TK, Zerbe A, Brittain K, Lesosky M, Hsiao NY, et al. Integration of postpartum healthcare services for HIV-infected women and their infants in South Africa: A randomised controlled trial. PLoS Med. 2018;15(3):e1002547.

14. Heffron R, Donnell D, Kiarie J, Rees H, Ngure K, Mugo N, et al. A prospective study of the effect of pregnancy on CD4 counts and plasma HIV-1 RNA concentrations of antiretroviral-naive HIV-1-infected women. J Acquir Immune Defic Syndr. 2014;65(2):231-6.

15. Walmsley SL, Antela A, Clumeck N, Duiculescu D, Eberhard A, Gutierrez F, et al. Dolutegravir plus abacavir-lamivudine for the treatment of HIV-1 infection. N Engl J Med. 2013;369(19):1807-18.

16. Paton NI, Kityo C, Hoppe A, Reid A, Kambugu A, Lugemwa A, et al. Assessment of second-line antiretroviral regimens for HIV therapy in Africa. N Engl J Med. 2014;371(3):234-47.

17. Losina E, Yazdanpanah Y, Deuffic-Burban S, Wang B, Wolf LL, Messou E, et al. The independent effect of highly active antiretroviral therapy on severe opportunistic disease incidence and mortality in HIV-infected adults in Côte d'Ivoire. Antiviral Therapy. 2007;12(4):543--51.

18. Ross EL, Weinstein MC, Schackman BR, Sax PE, Paltiel AD, Walensky RP, et al. The clinical role and cost-effectiveness of long-acting antiretroviral therapy. Clin Infect Dis. 2015;60(7):1102-10.

19. Currier JS, Britto P, Hoffman RM, Brummel S, Masheto G, Joao E, et al. Randomized trial of stopping or continuing ART among postpartum women with pre-ART CD4 >/= 400 cells/mm3. PLoS One. 2017;12(5):e0176009.

20. Mellors JW, Munoz A, Giorgi JV, Margolick JB, Tassoni CJ, Gupta P, et al. Plasma viral load and CD4+ lymphocytes as prognostic markers of HIV-1 infection. Ann Intern Med. 1997;126(12):946--54.

21. Rotheram-Borus MJ, Tomlinson M, Scheffler A, Le Roux IM. Re-engagement in HIV care among mothers living with HIV in South Africa over 36 months post-birth. AIDS. 2015;29(17):2361-2.

22. Chi BH, Yiannoutsos CT, Westfall AO, Newman JE, Zhou J, Cesar C, et al. Universal definition of loss to follow-up in HIV treatment programs: a statistical analysis of 111 facilities in Africa, Asia, and Latin America. PLoS Med. 2011;8(10):e1001111.

23. Danel C, Moh R, Chaix ML, Gabillard D, Gnokoro J, Diby CJ, et al. Two-months-off, four-months-on antiretroviral regimen increases the risk of resistance, compared with continuous therapy: a randomized trial involving West African adults. J Infect Dis. 2009;199(1):66-76.

24. Haas AD, Tenthani L, Msukwa MT, Tal K, Jahn A, Gadabu OJ, et al. Retention in care during the first 3 years of antiretroviral therapy for women in Malawi's option B+ programme: an observational cohort study. Lancet HIV. 2016;3(4):e175-82.

25. Meloni ST, Chang CA, Eisen G, Jolayemi T, Banigbe B, Okonkwo PI, et al. Long-term outcomes on antiretroviral therapy in a large scale-up program in Nigeria. PLoS One. 2016;11(10):e0164030.

26. South Africa Department of Health. National consolidated guidelines for the prevention of mother-to-child transmission of HIV (PMTCT) and the management of HIV in children, adolescents, and adults. <http://www.sahivsoc.org/Files/ART%20Guidelines%2015052015.pdf2015>.

27. Mandelbrot L, Tubiana R, Le Chenadec J, Dollfus C, Faye A, Pannier E, et al. No perinatal HIV-1 transmission from women with effective antiretroviral therapy starting before conception. Clin Infect Dis. 2015;61(11):1715-25.

28. Myer L, Phillips TK, McIntyre JA, Hsiao NY, Petro G, Zerbe A, et al. HIV viraemia and mother-to-child transmission risk after antiretroviral therapy initiation in pregnancy in Cape Town, South Africa. HIV Med. 2017;18(2):80-8.

29. Shapiro RL, Hughes MD, Ogwu A, Kitch D, Lockman S, Moffat C, et al. Antiretroviral regimens in pregnancy and breast-feeding in Botswana. N Engl J Med. 2010;362(24):2282-94.

30. Kesho Bora Study Group. Triple antiretroviral compared with zidovudine and single-dose nevirapine prophylaxis during pregnancy and breastfeeding for prevention of mother-to-child transmission of HIV-1 (Kesho Bora study): a randomised controlled trial. Lancet Infect Dis. 2011;11(3):171-80.

31. Cohan D, Natureeba P, Koss CA, Plenty A, Luwedde F, Mwesigwa J, et al. Efficacy and safety of lopinavir/ritonavir versus efavirenz-based antiretroviral therapy in HIV-infected pregnant Ugandan women. AIDS. 2015;29(2):183-91.

32. Perry ME, Taylor GP, Sabin CA, Conway K, Flanagan S, Dwyer E, et al. Lopinavir and atazanavir in pregnancy: comparable infant outcomes, virological efficacies and preterm delivery rates. HIV Med. 2016;17(1):28-35.

33. Peltier CA, Ndayisaba GF, Lepage P, van Griensven J, Leroy V, Pharm CO, et al. Breastfeeding with maternal antiretroviral therapy or formula feeding to prevent HIV postnatal mother-to-child transmission in Rwanda. AIDS. 2009;23(18):2415-23.

34. Ngoma MS, Misir A, Mutale W, Rampakakis E, Sampalis JS, Elong A, et al. Efficacy of WHO recommendation for continued breastfeeding and maternal cART for prevention of perinatal and postnatal HIV transmission in Zambia. J Int AIDS Soc. 2015;18:19352.

35. Kilewo C, Karlsson K, Massawe A, Lyamuya E, Swai A, Mhalu F, et al. Prevention of mother-to-child transmission of HIV-1 through breast-feeding by treating infants prophylactically with lamivudine in Dar es Salaam, Tanzania: the Mitra Study. J Acquir Immune Defic Syndr. 2008;48(3):315-23.

36. Thomas TK, Masaba R, Borkowf CB, Ndivo R, Zeh C, Misore A, et al. Triple-antiretroviral prophylaxis to prevent mother-to-child HIV transmission through breastfeeding--the Kisumu Breastfeeding Study, Kenya: a clinical trial. PLoS Med. 2011;8(3):e1001015.

37. Chigwedere P, Seage GR, Lee TH, Essex M. Efficacy of antiretroviral drugs in reducing mother-to-child transmission of HIV in Africa: a meta-analysis of published clinical trials. AIDS Res Hum Retroviruses. 2008;24(6):827-37.

38. Iliff PJ, Piwoz EG, Tavengwa NV, Zunguza CD, Marinda ET, Nathoo KJ, et al. Early exclusive breastfeeding reduces the risk of postnatal HIV-1 transmission and increases HIV-free survival. AIDS. 2005;19(7):699-708.

39. Fawzi W, Msamanga G, Spiegelman D, Renjifo B, Bang H, Kapiga S, et al. Transmission of HIV-1 through breastfeeding among women in Dar es Salaam, Tanzania. J Acquir Immune Defic Syndr. 2002;31(3):331--8.

40. Dabis F, Bequet L, Ekouevi DK, Viho I, Rouet F, Horo A, et al. Field efficacy of zidovudine, lamivudine and single-dose nevirapine to prevent peripartum HIV transmission. AIDS. 2005;19(3):309-18.

41. Kuhn L, Aldrovandi GM, Sinkala M, Kankasa C, Semrau K, Mwiya M, et al. Effects of early, abrupt weaning on HIV-free survival of children in Zambia. N Engl J Med. 2008;359(2):130-41.

42. Leroy V, Karon JM, Alioum A, Ekpini ER, Meda N, Greenberg AE, et al. Twenty-four month efficacy of a maternal short-course zidovudine regimen to prevent mother-to-child transmission of HIV-1 in West Africa. AIDS. 2002;16(4):631-41.

43. Thior I, Lockman S, Smeaton LM, Shapiro RL, Wester C, Heymann SJ, et al. Breastfeeding plus infant zidovudine prophylaxis for 6 months vs formula feeding plus infant zidovudine for 1 month to reduce mother-to-child HIV transmission in Botswana: a randomized trial: the Mashi Study. JAMA. 2006;296(7):794-805.

44. Petra Study Team. Efficacy of three short-course regimens of zidovudine and lamivudine in preventing early and late transmission of HIV-1 from mother to child in Tanzania, South Africa, and Uganda (Petra study): a randomised, double-blind, placebo-controlled trial. The Lancet. 2002;359(9313):1178-86.

45. Sherman G. Testing at birth - update from South Africa. 8th International Workshop on HIV Pediatrics; July 15, 2016; Durban, South Africa.

46. Bianchi F, Nzima V, Chadambuka A, Mataka A, Nyoni G, Ndayisaba G, Fassinou P, Machekano R, Sacks E, Bailey R, Alban R, Lemaire J, Cohn J, editor Comparing conventional to point-of-care (POC) early infant diagnosis (EID): Pre and post intervention data from a mutli-country evaluation. 9th Annual International AIDS Society Conference on HIV Science; 2017; Paris, France.

47. Cleary S, Chitha W, Jikwana S, Okorafor OA, Boulle A. Health systems trust: South African health review. 2005.

48. Thomas LS. Costing of HIV/AIDS services at a tertiary level hospital in Gauteng Province: Faculty of Health Sciences, University of Witwatersrand, South Africa; 2006 [cited 2014 May 6]. Available from: <http://wiredspace.wits.ac.za/handle/10539/2008>.

49. Holmes CB, Wood R, Badri M, Zilber S, Wang B, Maartens G, et al. CD4 decline and incidence of opportunistic infections in Cape Town, South Africa: implications for prophylaxis and treatment. J Acquir Immune Defic Syndr. 2006;42(4):464-9.

50. Clinton Health Access Initiative. Antiretroviral CHAI reference price list <https://clintonhealthaccess.org/content/uploads/2016/11/2016-CHAI-ARV-Reference-Price-List_FINAL.pdf2016> [cited 2017 September 27]. Available from: <https://clintonhealthaccess.org/content/uploads/2016/11/2016-CHAI-ARV-Reference-Price-List_FINAL.pdf>.

51. Doherty K, Essajee S, Penazzato M, Holmes C, Resch S, Ciaranello A. Estimating age-based antiretroviral therapy costs for HIV-infected children in resource-limited settings based on World Health Organization weight-based dosing recommendations. BMC Health Serv Res. 2014;14:201.

52. Koss CA, Natureeba P, Kwarisiima D, Ogena M, Clark TD, Olwoch P, et al. Viral suppression and retention in care up to 5 years after initiation of lifelong ART during pregnancy (Option B+) in rural Uganda. J Acquir Immune Defic Syndr. 2017;74(3):279-84.

53. Mancinelli S, Galluzzo CM, Andreotti M, Liotta G, Jere H, Sagno JB, et al. Virological response and drug resistance 1 and 2 years post-partum in HIV-infected women initiated on life-long antiretroviral therapy in Malawi. AIDS Res Hum Retroviruses. 2016;32(8):737-42.

54. Chetty T, Newell ML, Thorne C, Coutsoudis A. Viraemia before, during and after pregnancy in HIV-infected women on antiretroviral therapy in rural KwaZulu-Natal, South Africa, 2010-2015. Trop Med Int Health. 2018;23(1):79-91.

55. Eddy DM, Hollingworth W, Caro JJ, Tsevat J, McDonald KM, Wong JB, et al. Model transparency and validation: a report of the ISPOR-SMDM Modeling Good Research Practices Task Force--7. Value Health. 2012;15(6):843-50.

56. Goga AE, Dinh TH, Jackson DJ, Lombard C, Delaney KP, Puren A, et al. First population-level effectiveness evaluation of a national programme to prevent HIV transmission from mother to child, South Africa. J Epidemiol Community Health. 2015;69(3):240-8.

57. Goga AE JD, Lombard C, Ramokolo V, Ngandu N, , Sherman G PA, Doherty T, Bhardwaj S, Noveve N, , Ramraj T MV, Singh Y, Pillay, Dinh T for the SAPMTCTE study group. Highest risk of mother to child transmission of HIV or death in the first 6 months postpartum: results from 18 month follow-up of an HIV-exposed national cohort, South Africa. AIDS; Durban, South Africa, 2016.

58. Sherman GG, Mazanderani AH, Barron P, Bhardwaj S, Niit R, Okobi M, et al. Toward elimination of mother-to-child transmission of HIV in South Africa: how best to monitor early infant infections within the Prevention of Mother-to-Child Transmission Program. J Glob Health. 2017;7(1):010701.

59. Goga AE, Dinh TH, Jackson DJ, Lombard CJ, Puren A, Sherman G, et al. Population-level effectiveness of PMTCT Option A on early mother-to-child (MTCT) transmission of HIV in South Africa: implications for eliminating MTCT. J Glob Health. 2016;6(2):020405.

60. Arikawa S, Rollins N, Jourdain G, Humphrey J, Kourtis AP, Hoffman I, et al. Contribution of maternal ART and breastfeeding to 24-month survival in HIV-exposed uninfected children: an individual pooled analysis of African and Asian studies. Clin Infect Dis. 2017.

61. Cournil A, Van de Perre P, Cames C, de Vincenzi I, Read JS, Luchters S, et al. Early infant feeding patterns and HIV-free survival: findings from the Kesho-Bora trial (Burkina Faso, Kenya, South Africa). Pediatr Infect Dis J. 2015;34(2):168-74.

62. Ciaranello AL, Leroy V, Rusibamayila A, Freedberg KA, Shapiro R, Engelsmann B, et al. Individualizing the WHO HIV and infant feeding guidelines: optimal breastfeeding duration to maximize infant HIV-free survival. AIDS. 2014;28 Suppl 3:S287-99.

63. Woods B, Revill P, Sculpher M, and Claxton K. Country-level cost-effectiveness thresholds: Initial estimates and the need for further research. Centre for Health Economics, University of York, UK, 2015.

64. Robinson LA, Hammitt JK, Chang AY, Resch S. Understanding and improving the one and three times GDP per capita cost-effectiveness thresholds. Health Policy Plan. 2017;32(1):141-5.

65. Meyer-Rath G, van Rensburg C, Larson B, Jamieson L, Rosen S. Revealed willingness-to-pay versus standard cost-effectiveness thresholds: Evidence from the South African HIV Investment Case. PLoS One. 2017;12(10):e0186496.

66. Marseille E, Larson B, Kazi DS, Kahn JG, Rosen S. Thresholds for the cost-effectiveness of interventions: alternative approaches. Bull World Health Organ. 2015;93(2):118-24.
