# Supplementary figures and images for: Cost-effectiveness of integrating postpartum antiretroviral therapy and infant care into maternal & child health services in South Africa
Source: PLoS One. 2019 Nov 15;14(11):e0225104. doi: 10.1371/journal.pone.0225104 (PMC6857940; doi:10.1371/journal.pone.0225104)

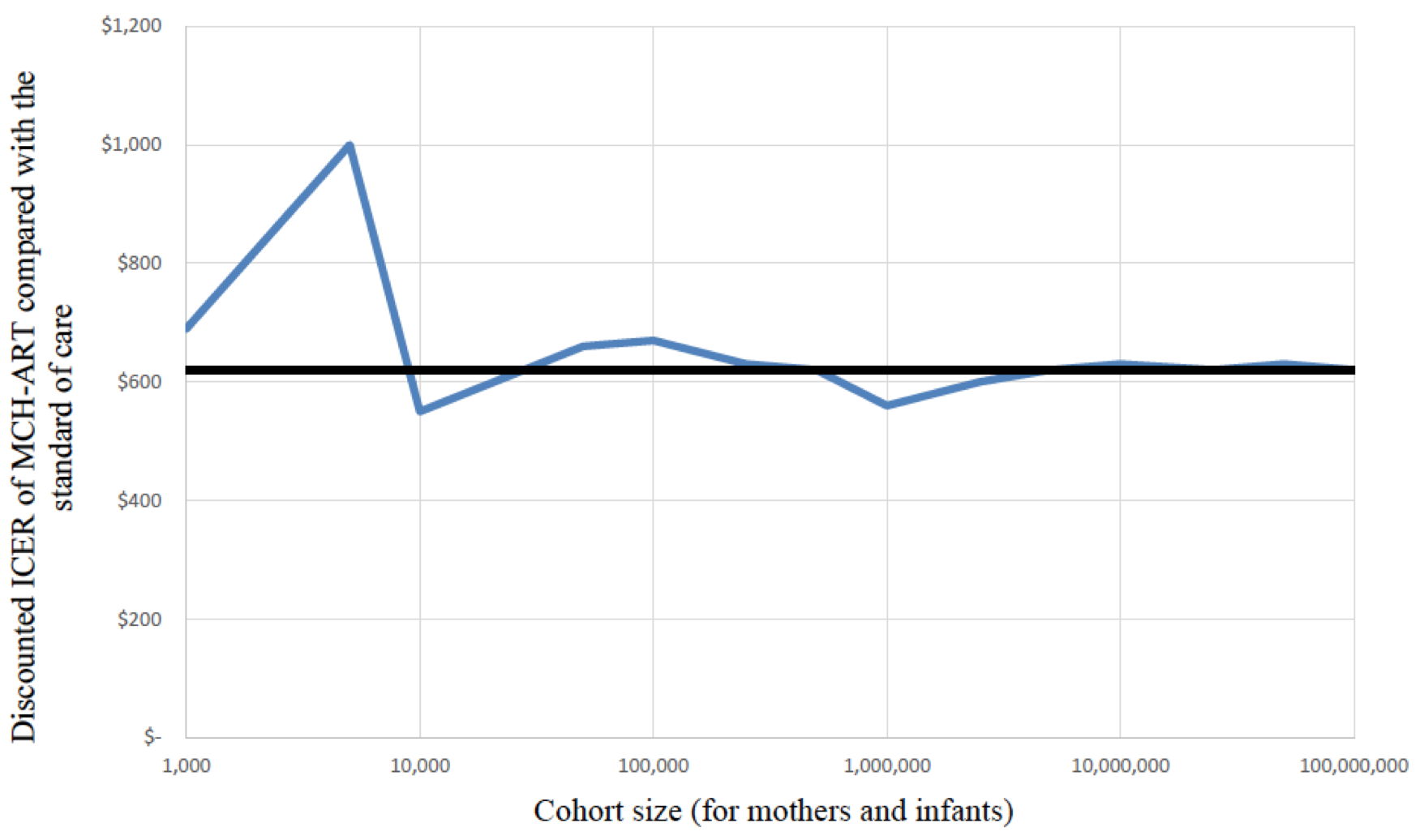

Supplement: S1 Fig — The discounted ICER (blue line) converged at cohort sizes (horizontal axis) of 10 million and greater for base case maternal and pediatric simulations for SOC and MCH-ART. Therefore, we chose a cohort size of 10 million for all simulations to produce stable per-person estimates. (TIFF) [file pone.0225104.s003.tiff]

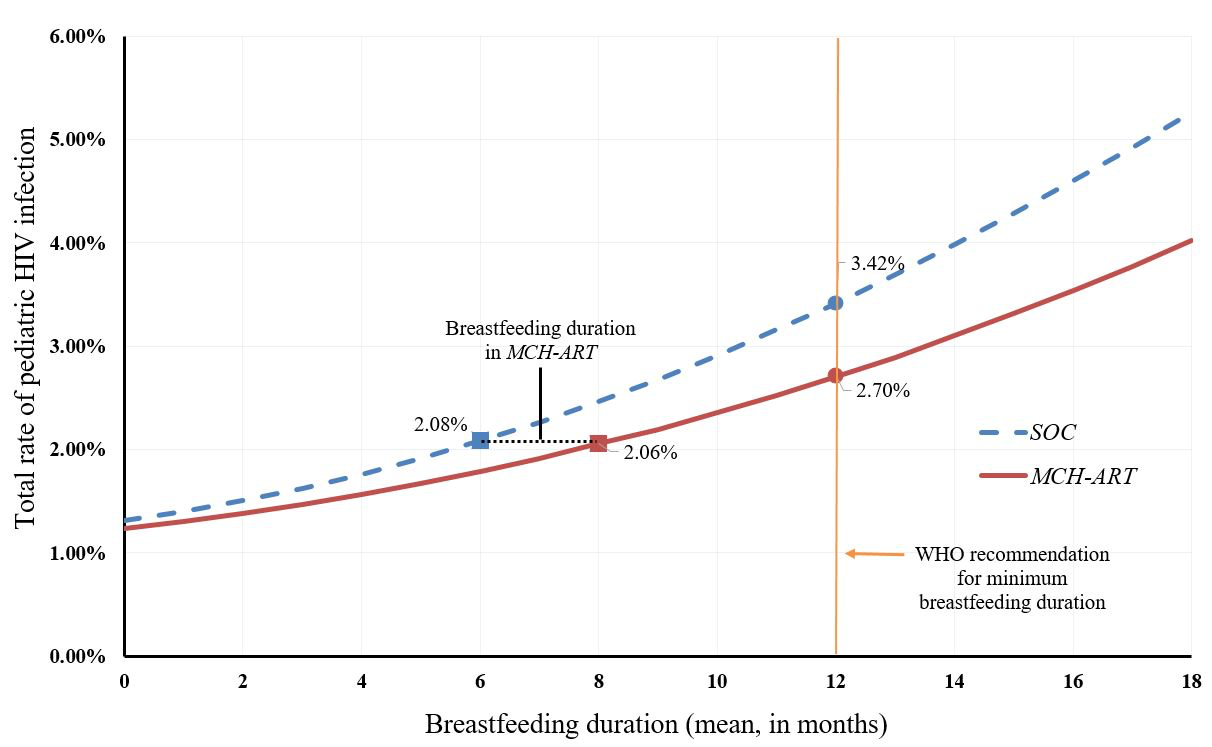

Supplement: S2 Fig — Projected pediatric HIV infection rates for the SOC and MCH-ART base case duration of breastfeeding (blue and red dots) and 12-month duration of breastfeeding (blue and red squares) are shown. (TIFF) [file pone.0225104.s004.tiff]

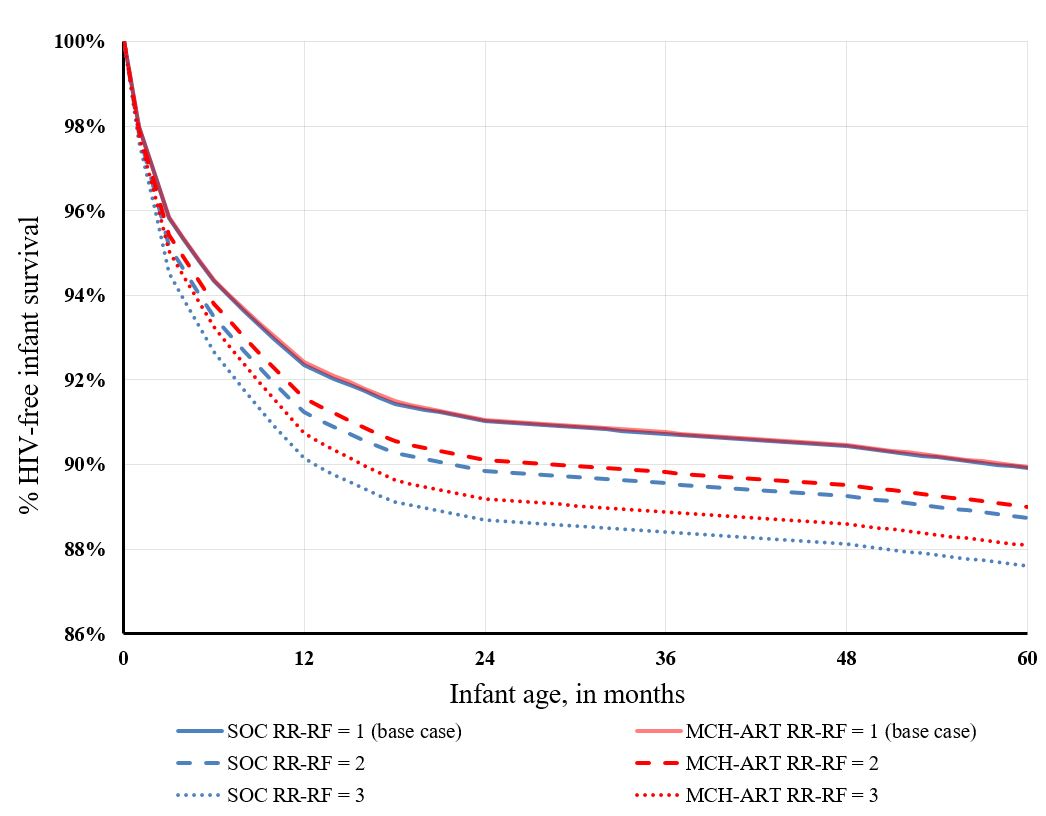

Supplement: S3 Fig — Base case (RR-RF = 1) assumes no increased relative risk of mortality with replacement feeding as compared to breastfeeding. (TIFF) [file pone.0225104.s005.tiff]
