# Supplementary material for: Cost-effectiveness of integrating postpartum antiretroviral therapy and infant care into maternal & child health services in South Africa
Source: PLoS One. 2019 Nov 15;14(11):e0225104. doi: 10.1371/journal.pone.0225104 (PMC6857940; doi:10.1371/journal.pone.0225104)
Supplement: S1 Table — (DOCX) [file pone.0225104.s006.docx]

| **S1 Table.** **Input parameters for a simulation model of women with HIV and their children in South Africa** | | | | |
| --- | --- | --- | --- | --- |
| **I. Clinical input parameters** | | | | |
| **Ia. Maternal cohort characteristics** | | | | |
| **Variable** | **Base case value** | | **Range examined** | **Data sources** |
| Age, mean (SD), years | 28.6 (5.4) | | 22-34 | [1] |
| Pre-ART CD4 count, median [IQR] | 354 [248, 517] | | 250-550 |  |
| Time on ART prior to delivery, median [IQR], months | 4 [3,6] | | 0-7 |  |
| Mothers on ART at delivery, % | 100 | | 0-100 |  |
| Mothers with HIV RNA <50 copies/mL at delivery, % | 76 | | 50-100 |  |
| Distribution of initial HIV RNA, % total |  | |  | [2] |
| >100,000 copies/mL | 10 | |  |  |
| 30,001-100,000 copies/mL | 25 | |  |  |
| 10,001-30,000 copies/mL | 13 | |  |  |
| 3,001-10,000 copies/mL | 24 | |  |  |
| 501-3,000 copies/mL | 18 | |  |  |
| 20-500 copies/mL | 6 | |  |  |
| < 20 copies/mL | 4 | |  |  |
| Frequency of monitoring while in care, months |  | |  |  |
| CD4 test interval, lifelong | 12 | | 0 after CD4 >350 to every 12 mo. lifelong | [3] |
| HIV RNA viral load test interval, <12 months on ART | 3 | | 1-12 |  |
| HIV RNA viral load test interval, >12 months on ART | 12 | | 6-24 |  |
| Care cascade characteristics during intervention period | SOC | MCH-ART |  |  |
| Retained in HIV care at 1 year, % | 71 | 81 | 60-100 | [1] |
| Retained in care and virologically suppressed (HIV RNA <50 copies/mL) at 1 year (of total starting cohort), % | 49 | 67 | 49-75 |  |
| Loss to follow-up after 12 months postpartum, %/mo. |  | |  |  |
| ART adherence <85%^a^ | 0.63 | | 0-3x | [4] |
| ART adherence >95%^a^ | 0.32 | | 0-3x |  |
| Return to care |  | |  |  |
| Probability after 6 months, monthly, % | 1.30 | | 0-5 | [5] |
| Probability upon WHO stage 3/4 OD, % | 50 | | 0-100 | Assumption |
| Likelihood of re-suppression on prior ART when returning to care, % | 96 | | 90-100 | [6] |
| **Ib. Pediatric cohort characteristics** | | | | |
| **Variable** | **Base case value** | | **Range examined** | **Data sources** |
| Age, mean (SD), months | 0 (0) | |  | Assumption |
| Mothers with CD4<350 before ART, % | 49 | | 10-90 | [1] |
| Monthly loss to follow-up after ART initiation, % | 0.20 | | 0-5x | [7, 8] |

| **Ic. Peri- and postnatal transmission parameters** | | | | | |
| --- | --- | --- | --- | --- | --- |
| **Variable** | **Base case value** | | | **Range examined** | **Data sources** |
| Intrauterine (IU)/intrapartum (IP) HIV transmission –  (60% IU, 40% IP, one-time risk), % |  | |  |  |  |
| On ART - suppressed (HIV RNA <50 copies/mL) | 0.44 | | | 0.00-0.88 | [9-14] |
| On ART - not suppressed (HIV RNA >50 copies/mL) | 2.57 | | | 0.00-10.00 | [9-14] |
| Not on ART (for sensitivity analyses), range by maternal CD4 | 17-27 | | | N/A | [15-20] |
| Postnatal transmission (monthly risk during breastfeeding), %/mo. |  | | |  |  |
| On ART- suppressed (HIV RNA <50 copies/mL) | 0.05 | | | 0.00-0.10 | [11] [13, 21, 22] |
| On ART - not suppressed (HIV RNA >50 copies/mL) | 0.21 | | | 0.00-1.20 | [11] [13, 21-24] |
| Not on ART, range by maternal CD4 |  | | |  |  |
| Exclusive breastfeeding | 0.24-0.76 | | | 0.25-2x | [15-20, 25, 26] |
| Mixed or complementary breastfeeding | 0.40-1.28 | | | 0.25-2x | [15-20, 25, 26] |
|  | SOC | | MCH-ART |  |  |
| Breastfeeding practices |  | |  |  | [1] |
| Breastfeeding duration, mean (SD), months | 6 (6) | | 8 (6) | 0-18 |  |
| Exclusive breastfeeding, % | 71 | | 77 | 0-100 |  |
| Mixed breastfeeding, % | 29 | | 23 | 0-100 |  |
| On ART, virologically suppressed during breastfeeding, HIV RNA <50 copies/mL (range by # months postpartum), % |  | |  |  |  |
| Months 0-6 postpartum | 62-81 | | 74-81 | 0.1-2x difference |  |
| Months 7-12 postpartum | 42-60 | | 78-80 | 0.1-2x difference |  |
| >12 months postpartum | 28-40 | | 44-76 | 0.1-2x difference |  |
| On ART, not suppressed during breastfeeding, HIV RNA >50 copies/mL (range by # months postpartum), % |  | | |  |  |
| Months 0-6 postpartum | 10-24 | | 8-24 | 0.1-2x difference |  |
| Months 7-12 postpartum | 24-33 | | 8-10 | 0.1-2x difference |  |
| >12 months postpartum | 23-31 | | 9-18 | 0.1-2x difference |  |
| **Id. Early infant diagnosis parameters** | | | | | |
| **Variable** | **Base case value** | | | **Range examined** | **Data sources** |
| Probability of infant having an HIV test, % | SOC | MCH-ART | |  |  |
| Birth test (initial NAT, confirmatory NAT) | 73 | 73 | | 0-100 | [27] |
| 6-10-week test (initial NAT, confirmatory NAT) | 78 | 82 | | 0-100 | [1] |
| 18-month test (initial POC RDT, confirmatory NAT) | 71 | 81 | | 0-100 | Assumption based on [1] |
| Probability of linking to care/ART after diagnosis, % | 71 | | | 0-100 | [28] |
| Delay between NAT test and result receipt, mean months (SD) | 1 (0) | | | 0-3 | [29] |
| Delay between POC test and result receipt, mean months (SD) | 0 (0) | | | 0-3 | [29] |
|  |  | | |  |  |

| **Id. Early infant diagnosis parameters (cont.)** | | | |
| --- | --- | --- | --- |
| **Variable** | **Base case value** | **Range examined** | **Data sources** |
| NAT characteristics (initial or confirmatory) |  |  |  |
| Sensitivity for IU/IP infection (by age), % | Month 1: 0, Later months: 99.40 | 90-100 | [30] |
| Sensitivity for PP infection (by age), % | Month of infection: 0, Later: 99.40 | 90-100 |  |
| Specificity, % | 99.60 | 90-100 |  |
| Point-of-care RDT for EID (18-month test) |  |  |  |
| Sensitivity for IU/IP infection (by age), % | Month 1: 0, Later months: 100.00 | 90-100 | [31] |
| Sensitivity for PP infection (by age), % | Month of infection: 0, Later: 100.00 | 90-100 |  |
| Specificity, % | 94.60 | 90-100 |  |
| Point-of-care RDT after presentation with OI (after 12 months old) |  |  |  |
| Sensitivity for IU/IP infection (by age), % | 98.20 (all ages) | 90-100 | [32] |
| Sensitivity for PP infection (by age), % | Month of infection: 0, Later: 98.20 | 90-100 |  |
| Specificity, % | 99.00 | 90-100 |  |
| **Ie. Natural history of disease** | | | |
| **Variable** | **Base case value** | **Range examined** | **Data sources** |
| **Natural history of HIV-infection (in absence of ART)** |  |  |  |
| CD4% at infection, mean (SD) | 45 (10) |  | [33] |
| Mean monthly decrease in CD4%, <60 months old, by age, % |  |  |  |
| <3 months of age, IU/IP infections only | 4.00 |  | [33] |
| 3-59 months of age, IU/IP infections only | 0.50 |  |  |
| 0-59 months of age, PP infections only | 0.50 |  |  |
| Mean monthly CD4 decline, >60 months old, range by HIV RNA, CD4 cells/µL | 3.03-6.38 |  | [33, 34] |
| Monthly risk of clinical events (range by CD4%) <60 months old, % |  |  |  |
| WHO Stage 3 event (except tuberculosis) | 3.29-11.61 |  | [35] |
| WHO Stage 4 event (except tuberculosis) | 1.39-6.35 |  |  |
| Tuberculosis (any body site) | 0.52-3.79 |  |  |

| **Ie. Natural history of disease (cont.)** | | | |
| --- | --- | --- | --- |
| **Variable** | **Base case value** | **Range examined** | **Data sources** |
| Monthly risk of clinical events (range by CD4) >60 months old, % |  |  |  |
| Mild fungal infection | 1.76-3.14 |  | [36] |
| Visceral bacterial infection | 0.04-0.71 |  |  |
| WHO Stage 3 or 4 visceral disease | 0.03-1.43 |  |  |
| WHO Stage 3 or 4 mucocutaneous disease | 0.03-2.26 |  |  |
| Other WHO Stage 3 or 4 disease | 0.02-0.73 |  |  |
| Other severe disease | 0.20-1.67 |  |  |
| Other mild disease | 2.39 |  |  |
| Tuberculosis (any body site) | 0.03-1.74 |  |  |
| Risk of death within 30 days of clinical event <60 months old, % |  |  |  |
| After WHO Stage 3 or 4 event | 13.45 |  | [33, 35] |
| After tuberculosis event | 11.10 |  |  |
| Risk of death within 30 days of clinical event > 60 months old, % |  | |  |
| Mild fungal infection | 0.54 |  | [36] |
| Visceral bacterial infection | 2.94 |  |  |
| WHO Stage 3 or 4 visceral disease | 9.21 |  |  |
| WHO Stage 3 or 4 mucocutaneous disease | 2.38 |  |  |
| Other WHO Stage 3 or 4 disease | 20.00 |  |  |
| Other severe disease | 6.67 |  |  |
| Other mild disease | 0.39 |  |  |
| Tuberculosis (any body site) | 1.82 |  |  |
| Monthly risk of HIV-related death (range by age, CD4%/CD4, and history of prior OI), % | 0.16-40.80 |  | [33, 35, 36] |
| Monthly risk of non-AIDS related mortality (range by age in yearly intervals, sex), % |  |  |  |
| <5 years of age | 0.02-0.03 |  | [37] |
| 5-12 years of age | 0.00-0.01 |  |  |
| 13-18 years of age | 0.00-0.01 |  |  |
| >18 years of age | 0.00-1.40 |  |  |
| **Natural history of HIV-exposed, uninfected children** |  |  |  |
| Monthly risk of infant mortality among HIV-exposed, uninfected infants), % |  |  |  |
| 0-2 months | 1.01 |  | [38, 39] |
| 3-5 months | 0.41 |  |  |
| 6-11 months | 0.28 |  |  |
| 12-17 months | 0.14 |  |  |
| 18-23 months | 0.07 |  |  |

| **If. Antiretroviral therapy and co-trimoxazole prophylaxis** | | | | |
| --- | --- | --- | --- | --- |
| **Variable** | **Variable** | | **Variable** | **Variable** |
| ART efficacy (virologically suppressed at 24 weeks) - children | ABC/3TC/LPV/r (1^st^ line) | AZT/3TC/EFV (2^nd^ line) |  |  |
| Ages 0-59 months, % | 91 | 75 | 70-100 | [40, 41] |
| Ages 60+ months, % | 82 | 82 | 70-100 | [42] |
| Probability of virologic failure after initial suppression - children |  | |  |  |
| Ages 0-59 months, any regimen, per month, % | 0.91 | | 0.5-2x | [40, 41] |
| Ages 60+ months, any regimen, per month, % | 0.72 | | 0.5-2x | [42] |
|  | TDF/FTC/EFV (1^st^ line) | AZT/3TC/LPV/r (2^nd^ line) |  |  |
| ART efficacy (% virologically suppressed at 48 weeks) - adults | 95 | 89 | 80-100 | [1, 43] |
| Probability of virologic failure after initial suppression – adults |  |  |  |  |
| ART adherence <85%, per month, %^a^ | 10.80 | 7.00 | 0.5-2x | [1, 44-46] |
| ART adherence >95%, per month, %^a^ | 0.02 | 0.02 | 0.5-2x | [1, 44-46] |
| Probability of experiencing toxicity while on ART – adults, % |  |  |  |  |
| Minor toxicity, one-time risk | 17.10 | 19.00 | 0.5-2x | [13] |
| Major toxicity, one-time risk | 1.16 | 2.18 | 0.5-2x | [11, 47] |
| Relative risk reduction for patients on ART, % |  | |  |  |
| Risk of opportunistic disease (age 0-13) | 85 | | 0-100 | [33] |
| Risk of opportunistic disease (age 13+) | 32 | | 0-100 | [48] |
| Mortality risk (age 0-13) | 90 | | 0-100 | [33] |
| Mortality risk (age 13+), range by CD4 | 55-96 | | 0-100 | [48] |
| Reduction in risk of infection while on co-trimoxazole prophylaxis, % |  | |  |  |
| Mild fungal infection | -46.37 | | 0-1.25x | [49, 50] |
| Mild bacterial diseases | 48.79 | | 0-1.25x |  |
| Invasive bacterial diseases | 49.81 | | 0-1.25x |  |
| WHO stage 3-4 visceral diseases | 17.86 | | 0-1.25x |  |
| Other severe events | 17.88 | | 0-1.25x |  |
| Toxicity of co-trimoxazole prophylaxis, one-time risk, % |  | |  |  |
| Minor toxicity | 16.67 | | 0.5-2x | [49, 50] |
| Major toxicity | 6.50 | | 0.5-2x |  |
| **II. Economic model input parameters** | | | | |
| **IIa. Laboratory and medication costs** | | | | |
| **Variable** | **2016 USD** | | **Range examined** | **Data sources** |
| Maternal ART (per month) |  | |  |  |
| 1^st^ line (TDF/FTC/EFV) | 9 | | 5-18 | [51] |
| 2^nd^ line (AZT/3TC/LPV/r) | 27 | | 14-54 |  |
| Pediatric ART (range by age and weight, per month) |  | |  |  |
| 1^st^ line (ABC/3TC/LPV/r) | 21-44 | | 0.5-2x | [51, 52] |
| 2^nd^ line (AZT/3TC/EFV) | 10-25 | | 0.5-2x |  |
|  |  | |  |  |

| **IIa. Laboratory and medication costs (cont.)** | | | |
| --- | --- | --- | --- |
| **Variable** | **2016 USD** | **Range examined** | **Data sources** |
| Maternal co-trimoxazole prophylaxis (per month) | 4 | 2-8 | [53] |
| CD4 assay | 12 | 6-24 | [54] |
| HIV RNA (viral load) test | 22 | 11-44 | [54] |
| Early infant diagnosis tests |  |  |  |
| DNA NAT assay (0-9 months) | 24 | 12-48 | [54] |
| Negative NAT result-return cost | 1 | 0-2 | [55] |
| Positive NAT result-return cost | 2 | 1-4 | [55] |
| HIV RDT (>9 months) | 3 | 2-6 | [54] |
| **IIb. Healthcare costs** | | | |
| **Variable** | **2016 USD** | **Range examined** | **Data sources** |
| Care for acute OD (per event), <5 years old: |  |  |  |
| WHO stage 3 | 808 | 0.5-2x | [56, 57] |
| WHO stage 4 | 1420 | 0.5-2x |  |
| Tuberculosis | 1077 | 0.5-2x |  |
| Care for acute OD (per event), >5 years old: |  |  |  |
| WHO stage 3-4 visceral | 679 | 0.5-2x | [36, 56] |
| WHO stage 3-4 non-visceral | 435 | 0.5-2x |  |
| WHO stage 3-4 non-specific | 360 | 0.5-2x |  |
| Bacterial infection | 639 | 0.5-2x |  |
| Mild fungal infection | 300 | 0.5-2x |  |
| Tuberculosis | 642 | 0.5-2x |  |
| Other mild infection | 202 | 0.5-2x |  |
| Other severe infection | 433 | 0.5-2x |  |
| Terminal care, last month of life | 507 | 0.5-2x | [36, 56] |
| Maternal drug toxicities costs |  |  |  |
| Major drug toxicity | 1390 | 0.5-2x | [50, 58] |
| Minor drug toxicity | 21 | 0.5-2x |  |
| Routine care costs (per month) |  |  |  |
| CD4 >500 cells/µL (adults) or CD4 >35% (children) | 17 | 0.5-2x | [36, 56] |
| CD4 351-500 cells/µL (adults) or CD4 25-35% (children) | 22 | 0.5-2x |  |
| CD4 201-350 cells/µL (adults) or CD4 15-25% (children) | 26 | 0.5-2x |  |
| CD4 51-200 cells/µL (adults) or CD4 5-15% (children) | 57 | 0.5-2x |  |
| CD4 <50 cells/µL (adults) or CD4 <5% (children) | 129 | 0.5-2x |  |
| 12-month postpartum healthcare costs |  |  |  |
| SOC | 50 |  |  |
| MCH-ART | 69 | 35-138 |  |
| **SD:** Standard deviation; **ANC:** antenatal care; **ART:** antiretroviral therapy; **IQR:** interquartile range; **RNA:** ribonucleic acid; **SOC:** standard of care strategy; **LTFU:** lost to follow-up; **WHO:** World Health Organization; **OD:** opportunistic disease; **NAT**: nucleic acid test; **POC:** point-of-care; **RDT:** rapid diagnostic test; **ABC:** abacavir; **3TC:** lamivudine; **LPV:** lopinavir; **r:** ritonavir; **AZT:** azidothymidine; **EFV:** efavirenz; **TDF:** tenofovir disoproxil fumarate; **FTC:** emtricitabine; **USD:** United States dollars. | | | |

^a^ Values from 85% to 95% were interpolated. For more information, see: Ross E et al. [59].

^b^ No 18-month EID testing uptake data were available. Therefore, we assumed that EID testing rates would mirror maternal retention in care at 12 months as observed in the MCH-ART trial.

**REFERENCES:**

1. Myer L, Phillips TK, Zerbe A, Brittain K, Lesosky M, Hsiao NY, et al. Integration of postpartum healthcare services for HIV-infected women and their infants in South Africa: A randomised controlled trial. PLoS Med. 2018;15(3):e1002547.

2. Myer L, Phillips TK, Hsiao NY, Zerbe A, Petro G, Bekker LG, et al. Plasma viraemia in HIV-positive pregnant women entering antenatal care in South Africa. J Int AIDS Soc. 2015;18(1):20045.

3. South Africa Department of Health. National consolidated guidelines for the prevention of mother-to-child transmission of HIV (PMTCT) and the management of HIV in children, adolescents, and adults. <http://www.sahivsoc.org/Files/ART%20Guidelines%2015052015.pdf2015>.

4. Haas AD, Tenthani L, Msukwa MT, Tal K, Jahn A, Gadabu OJ, et al. Retention in care during the first 3 years of antiretroviral therapy for women in Malawi's option B+ programme: an observational cohort study. Lancet HIV. 2016;3(4):e175-82.

5. Rotheram-Borus MJ, Tomlinson M, Scheffler A, Le Roux IM. Re-engagement in HIV care among mothers living with HIV in South Africa over 36 months post-birth. AIDS. 2015;29(17):2361-2.

6. Danel C, Moh R, Chaix ML, Gabillard D, Gnokoro J, Diby CJ, et al. Two-months-off, four-months-on antiretroviral regimen increases the risk of resistance, compared with continuous therapy: a randomized trial involving West African adults. J Infect Dis. 2009;199(1):66-76.

7. Ciaranello AL, Chang Y, Margulis AV, Bernstein A, Bassett IV, Losina E, et al. Effectiveness of pediatric antiretroviral therapy in resource-limited settings: a systematic review and meta-analysis. Clin Infect Dis. 2009;49(12):1915-27.

8. Fox MP, Rosen S. Systematic review of retention of pediatric patients on HIV treatment in low and middle-income countries 2008-2013. AIDS. 2015;29(4):493-502.

9. Mandelbrot L, Tubiana R, Le Chenadec J, Dollfus C, Faye A, Pannier E, et al. No perinatal HIV-1 transmission from women with effective antiretroviral therapy starting before conception. Clin Infect Dis. 2015;61(11):1715-25.

10. Myer L, Phillips TK, McIntyre JA, Hsiao NY, Petro G, Zerbe A, et al. HIV viraemia and mother-to-child transmission risk after antiretroviral therapy initiation in pregnancy in Cape Town, South Africa. HIV Med. 2017;18(2):80-8.

11. Shapiro RL, Hughes MD, Ogwu A, Kitch D, Lockman S, Moffat C, et al. Antiretroviral regimens in pregnancy and breast-feeding in Botswana. N Engl J Med. 2010;362(24):2282-94.

12. Kesho Bora Study Group. Triple antiretroviral compared with zidovudine and single-dose nevirapine prophylaxis during pregnancy and breastfeeding for prevention of mother-to-child transmission of HIV-1 (Kesho Bora study): a randomised controlled trial. Lancet Infect Dis. 2011;11(3):171-80.

13. Cohan D, Natureeba P, Koss CA, Plenty A, Luwedde F, Mwesigwa J, et al. Efficacy and safety of lopinavir/ritonavir versus efavirenz-based antiretroviral therapy in HIV-infected pregnant Ugandan women. AIDS. 2015;29(2):183-91.

14. Perry ME, Taylor GP, Sabin CA, Conway K, Flanagan S, Dwyer E, et al. Lopinavir and atazanavir in pregnancy: comparable infant outcomes, virological efficacies and preterm delivery rates. HIV Med. 2016;17(1):28-35.

15. Fawzi W, Msamanga G, Spiegelman D, Renjifo B, Bang H, Kapiga S, et al. Transmission of HIV-1 through breastfeeding among women in Dar es Salaam, Tanzania. J Acquir Immune Defic Syndr. 2002;31(3):331--8.

16. Petra Study Team. Efficacy of three short-course regimens of zidovudine and lamivudine in preventing early and late transmission of HIV-1 from mother to child in Tanzania, South Africa, and Uganda (Petra study): a randomised, double-blind, placebo-controlled trial. The Lancet. 2002;359(9313):1178-86.

17. Leroy V, Karon JM, Alioum A, Ekpini ER, Meda N, Greenberg AE, et al. Twenty-four month efficacy of a maternal short-course zidovudine regimen to prevent mother-to-child transmission of HIV-1 in West Africa. AIDS. 2002;16(4):631-41.

18. Chigwedere P, Seage GR, Lee TH, Essex M. Efficacy of antiretroviral drugs in reducing mother-to-child transmission of HIV in Africa: a meta-analysis of published clinical trials. AIDS Res Hum Retroviruses. 2008;24(6):827-37.

19. Dabis F, Bequet L, Ekouevi DK, Viho I, Rouet F, Horo A, et al. Field efficacy of zidovudine, lamivudine and single-dose nevirapine to prevent peripartum HIV transmission. AIDS. 2005;19(3):309-18.

20. Thior I, Lockman S, Smeaton LM, Shapiro RL, Wester C, Heymann SJ, et al. Breastfeeding plus infant zidovudine prophylaxis for 6 months vs formula feeding plus infant zidovudine for 1 month to reduce mother-to-child HIV transmission in Botswana: a randomized trial: the Mashi Study. JAMA. 2006;296(7):794-805.

21. Peltier CA, Ndayisaba GF, Lepage P, van Griensven J, Leroy V, Pharm CO, et al. Breastfeeding with maternal antiretroviral therapy or formula feeding to prevent HIV postnatal mother-to-child transmission in Rwanda. AIDS. 2009;23(18):2415-23.

22. Ngoma MS, Misir A, Mutale W, Rampakakis E, Sampalis JS, Elong A, et al. Efficacy of WHO recommendation for continued breastfeeding and maternal cART for prevention of perinatal and postnatal HIV transmission in Zambia. J Int AIDS Soc. 2015;18:19352.

23. Kilewo C, Karlsson K, Massawe A, Lyamuya E, Swai A, Mhalu F, et al. Prevention of mother-to-child transmission of HIV-1 through breast-feeding by treating infants prophylactically with lamivudine in Dar es Salaam, Tanzania: the Mitra Study. J Acquir Immune Defic Syndr. 2008;48(3):315-23.

24. Thomas TK, Masaba R, Borkowf CB, Ndivo R, Zeh C, Misore A, et al. Triple-antiretroviral prophylaxis to prevent mother-to-child HIV transmission through breastfeeding--the Kisumu Breastfeeding Study, Kenya: a clinical trial. PLoS Med. 2011;8(3):e1001015.

25. Iliff PJ, Piwoz EG, Tavengwa NV, Zunguza CD, Marinda ET, Nathoo KJ, et al. Early exclusive breastfeeding reduces the risk of postnatal HIV-1 transmission and increases HIV-free survival. AIDS. 2005;19(7):699-708.

26. Kuhn L, Aldrovandi GM, Sinkala M, Kankasa C, Semrau K, Mwiya M, et al. Effects of early, abrupt weaning on HIV-free survival of children in Zambia. N Engl J Med. 2008;359(2):130-41.

27. Sherman G. Testing at birth - update from South Africa. 8th International Workshop on HIV Pediatrics; July 15, 2016; Durban, South Africa.

28. Hsiao NY, Stinson K, Myer L. Linkage of HIV-infected infants from diagnosis to antiretroviral therapy services across the Western Cape, South Africa. PLoS One. 2013;8(2):e55308.

29. Bianchi F, Nzima V, Chadambuka A, Mataka A, Nyoni G, Ndayisaba G, Fassinou P, Machekano R, Sacks E, Bailey R, Alban R, Lemaire J, Cohn J, editor Comparing conventional to point-of-care (POC) early infant diagnosis (EID): Pre and post intervention data from a mutli-country evaluation. 9th Annual International AIDS Society Conference on HIV Science; 2017; Paris, France.

30. Mallampati D, Ford N, Hannaford A, Sugandhi N, Penazzato M. Performance of virological testing for early infant diagnosis: A systematic review. J Acquir Immune Defic Syndr. 2017;75(3):308-14.

31. Smith ER, Sheahan AD, Heyderman RS, Miller WC, Wheeler S, Hudgens M, et al. Performance of HIV rapid tests among breastfeeding, Malawian infants. Pediatr Infect Dis J. 2017;36(4):405-11.

32. Buchanan AM, Nadjm B, Amos B, Mtove G, Sifuna D, Cunningham CK, et al. Utility of rapid antibody tests to exclude HIV-1 infection among infants and children aged <18 months in a low-resource setting. J Clin Virol. 2012;55(3):244-9.

33. Ciaranello AL, Morris BL, Walensky RP, Weinstein MC, Ayaya S, Doherty K, et al. Validation and calibration of a computer simulation model of pediatric HIV infection. PLoS One. 2013;8(12):e83389.

34. Mellors JW, Munoz A, Giorgi JV, Margolick JB, Tassoni CJ, Gupta P, et al. Plasma viral load and CD4+ lymphocytes as prognostic markers of HIV-1 infection. Ann Intern Med. 1997;126(12):946--54.

35. Ciaranello A, Lu Z, Ayaya S, Losina E, Musick B, Vreeman R, et al. Incidence of World Health Organization stage 3 and 4 events, tuberculosis and mortality in untreated, HIV-infected children enrolling in care before 1 year of age: an IeDEA (International Epidemiologic Databases To Evaluate AIDS) East Africa regional analysis. Pediatr Infect Dis J. 2014;33(6):623-9.

36. Holmes CB, Wood R, Badri M, Zilber S, Wang B, Maartens G, et al. CD4 decline and incidence of opportunistic infections in Cape Town, South Africa: implications for prophylaxis and treatment. J Acquir Immune Defic Syndr. 2006;42(4):464-9.

37. United Nations Population Division Department of Economic and Social Affairs. World Population Prospects (2008 revision).

38. Marston M, Becquet R, Zaba B, Moulton LH, Gray G, Coovadia H, et al. Net survival of perinatally and postnatally HIV-infected children: a pooled analysis of individual data from sub-Saharan Africa. Int J Epidemiol. 2011;40(2):385-96.

39. Becquet R, Marston M, Dabis F, Moulton LH, Gray G, Coovadia HM, et al. Children who acquire HIV infection perinatally are at higher risk of early death than those acquiring infection through breastmilk: a meta-analysis. PLoS One. 2012;7(2):e28510.

40. Violari A, Lindsey JC, Hughes MD, Mujuru HA, Barlow-Mosha L, Kamthunzi P, et al. Nevirapine versus ritonavir-boosted lopinavir for HIV-infected children. N Engl J Med. 2012;366(25):2380-9.

41. Palumbo P, Lindsey JC, Hughes MD, Cotton MF, Bobat R, Meyers T, et al. Antiretroviral treatment for children with peripartum nevirapine exposure. N Engl J Med. 2010;363(16):1510-20.

42. Barth RE, van der Loeff MF, Schuurman R, Hoepelman AI, Wensing AM. Virological follow-up of adult patients in antiretroviral treatment programmes in sub-Saharan Africa: a systematic review. Lancet Infect Dis. 2010;10(3):155-66.

43. Paton NI, Kityo C, Hoppe A, Reid A, Kambugu A, Lugemwa A, et al. Assessment of second-line antiretroviral regimens for HIV therapy in Africa. N Engl J Med. 2014;371(3):234-47.

44. Koss CA, Natureeba P, Kwarisiima D, Ogena M, Clark TD, Olwoch P, et al. Viral suppression and retention in care up to 5 years after initiation of lifelong ART during pregnancy (Option B+) in rural Uganda. J Acquir Immune Defic Syndr. 2017;74(3):279-84.

45. Mancinelli S, Galluzzo CM, Andreotti M, Liotta G, Jere H, Sagno JB, et al. Virological response and drug resistance 1 and 2 years post-partum in HIV-infected women initiated on life-long antiretroviral therapy in Malawi. AIDS Res Hum Retroviruses. 2016;32(8):737-42.

46. Chetty T, Newell ML, Thorne C, Coutsoudis A. Viraemia before, during and after pregnancy in HIV-infected women on antiretroviral therapy in rural KwaZulu-Natal, South Africa, 2010-2015. Trop Med Int Health. 2018;23(1):79-91.

47. Phillips T, Cois A, Remien RH, Mellins CA, McIntyre JA, Petro G, et al. Self-reported side effects and adherence to antiretroviral therapy in HIV-infected pregnant women under option B+: A prospective study. PLoS One. 2016;11(10):e0163079.

48. Losina E, Yazdanpanah Y, Deuffic-Burban S, Wang B, Wolf LL, Messou E, et al. The independent effect of highly active antiretroviral therapy on severe opportunistic disease incidence and mortality in HIV-infected adults in Côte d'Ivoire. Antiviral Therapy. 2007;12(4):543--51.

49. Yazdanpanah Y, Losina E, Anglaret X, Goldie SJ, Walensky RP, Weinstein MC, et al. Clinical impact and cost-effectiveness of co-trimoxazole prophylaxis in patients with HIV/AIDS in Côte d'Ivoire: a trial-based analysis. Aids. 2005;19(12):1299--308.

50. Anglaret X, Chene G, Attia A, Toure S, Lafont S, Combe P, et al. Early chemoprophylaxis with trimethoprim-sulphamethoxazole for HIV-1-infected adults in Abidjan, Côte d'Ivoire: a randomised trial. Cotrimo-CI Study Group. Lancet. 1999;353(9163):1463--8.

51. Clinton Health Access Initiative. Antiretroviral CHAI reference price list <https://clintonhealthaccess.org/content/uploads/2016/11/2016-CHAI-ARV-Reference-Price-List_FINAL.pdf2016> [cited 2017 September 27]. Available from: <https://clintonhealthaccess.org/content/uploads/2016/11/2016-CHAI-ARV-Reference-Price-List_FINAL.pdf>.

52. Doherty K, Essajee S, Penazzato M, Holmes C, Resch S, Ciaranello A. Estimating age-based antiretroviral therapy costs for HIV-infected children in resource-limited settings based on World Health Organization weight-based dosing recommendations. BMC Health Serv Res. 2014;14:201.

53. Central Medical Stores Swaziland. Personal communication. 2016.

54. Dr. Leigh Berrie at National Health Laboratory Services South Africa. Personal communication. 2016.

55. Bassett IV, Giddy J, Nkera J, Wang B, Losina E, Lu Z, et al. Routine voluntary HIV testing in Durban, South Africa: the experience from an outpatient department. J Acquir Immune Defic Syndr. 2007;46(2):181-6.

56. Cleary S, Chitha W, Jikwana S, Okorafor OA, Boulle A. Health systems trust: South African health review. 2005.

57. Thomas LS. Costing of HIV/AIDS services at a tertiary level hospital in Gauteng Province: Faculty of Health Sciences, University of Witwatersrand, South Africa; 2006 [cited 2014 May 6]. Available from: <http://wiredspace.wits.ac.za/handle/10539/2008>.

58. World Health Organization. WHO-CHOICE unit cost estimates for service delivery 2008. Available from: <http://www.who.int/choice/costs/en/>.

59. Ross EL, Weinstein MC, Schackman BR, Sax PE, Paltiel AD, Walensky RP, et al. The clinical role and cost-effectiveness of long-acting antiretroviral therapy. Clin Infect Dis. 2015;60(7):1102-10.
