# Supplementary material for: Cost-effectiveness of integrating postpartum antiretroviral therapy and infant care into maternal & child health services in South Africa
Source: PLoS One. 2019 Nov 15;14(11):e0225104. doi: 10.1371/journal.pone.0225104 (PMC6857940; doi:10.1371/journal.pone.0225104)
Supplement: S2 Table — (DOCX) [file pone.0225104.s007.docx]

**S2 Table. Sources of data and ranges for sensitivity analyses**

| **CEPAC Input parameters** | | | |
| --- | --- | --- | --- |
| **Point Estimate** | **Source** | **Uncertainty Range** | **Sensitivity Analysis**  **Range** |
| **Maternal Cohort Characteristics** | | | |
| **Age, mean** | | | |
| 28.6 years | Myer et al. *PLoS Med* 2018 [1] – MCH-ART trial: mean age 28.6 (5.4). Examined the range of mean age from one standard deviation above to one standard deviation below the mean age observed in the trial | n/a | 22-34 |
| **Pre-ART CD4 count, median** | | | |
| 354 cells/µL | Myer et al. *PLoS Med* 2018 [1] – MCH-ART trial: median pre-ART CD4 354 cells/µL (IQR: 248, 517). Examined median pre-ART CD4 from the lower to the upper limit of the IQR reported from trial. | n/a | 250-550 |
| **Women with HIV VL < 50 copies/mL at delivery, %** | | | |
| 76% | Myer et al. *PLoS Med* 2018 [1] – MCH-ART trial: 76%  Evaluated the following studies that reported virologic suppression (VS) rates at delivery for women initiated on ART during the index pregnancy in low-resource settings:  Myer et al. *HIV Med* 2017 [2] – South Africa: 73%, VS = VL <50 copies/mL  Shapiro et al. *NEJM* 2010 [3] – Botswana: 96% (NRTI), 93% (PI), and 94% (observational, on NVP + ZDV/3TC), VS = VL <400 copies/mL  Kesho Bora Study Group. *Lancet Infect Dis* 2011 [4] – Burkino Faso, Kenya, and South Africa: 64%, VS = VL <300 copies/mL  Cohan et al. *AIDS* 2015 [5] – Uganda: 97.6% (EFV) and 86.0% (LPV/r), VS = VL <400 copies/mL  Fowler et al. *NEJM* 2016 [6] – India, Malawi, South Africa, Tanzania, Uganda, Zambia, and Zimbabwe: 65-68% (ZDV/3TC/LPV/r), 57% (TDF/FTC/LPV/r), VS = <400 copies/mL  Chagomerana et al. *PLoS One* 2018 [7] – Malawi: 69%, VS = <40 copies/mL | 57-97.6% | 50-100 |
| **Months on maternal ART prior to delivery** | | | |
| 4 months | Myer et al. *PLoS Med* 2018 [1] – MCH-ART trial: median time on ART prior to delivery 4 months (IQR: 3, 5). We explored the range of 0-7 months, as some women present at delivery without antenatal care and others may present shortly after recognition of pregnancy (approx. 2 months into pregnancy). | 3-5 | 0-7 |
| **Background maternal return to care, %/mo.** | | | |
| 1.30%/mo. | Rotheram-Borus et al. *AIDS* 2015 [8] – Postpartum women in South Africa: 1.30%/mo. Used this as the base case point estimate, as this population most closely matches that examined in the MCH-ART trial.  Kaplan et al. *PLoS Med* 2017 [9] – Adult patients who visited ART clinics in Khayelitsha, South Africa: 33% of those who disengaged from care returned to ART care after 180 days over a 2 year window. When converted to a monthly probability, this equates to 1.66%/mo.  Bershetyn et al. *Clin Infect Dis* 2017 [10] – Adult patients in Uganda, Kenya, and Tanzania: 10.0% (95% CI, 9.1%-10.8%) of patients who were LTFU and were not selected for tracing returned to care within 1 year. Monthly probability = 0.87% (95% CI: 0.79%-0.95%) | 0.79-1.66 | 0-5 |

| **MCH-ART intervention parameters** | | | | |
| --- | --- | --- | --- | --- |
| **Postpartum women retained in HIV care at 1 year in MCH-ART** | | | | |
| 81% | Myer et al. *PLoS Med* 2018 [1] – MCH-ART trial: 81% of women in the MCH-ART intervention were retained in care at 12 months compared with 71% of women in the standard of care (SOC) arm. We ranged retention in MCH-ART from 71% (10% less than base case for MCH-ART, equal to SOC) to 91% (10% more than base case, doubling the absolute difference in retention observed between the arms of the MCH-ART trial).  Knettel et al. *JAIDS* 2018 [11] – Meta-analysis of maternal retention in care during pregnancy and postpartum in the Option B+ era from studies from Africa. 35 articles were included in the final review. The pooled estimate of retention at 12 months was 76.4% (95% CI: 69.0-83.1%). The lowest 12-month retention rate was reported in Mozambique at 42% (Llenas-Garcia et al. 2016 *Trop Med Int Health* [12]). However, no other estimate dropped below 60%. The highest 12-month retention rate was documented in Malawi at 97% (Kamuyango et al. *World J AIDS* 2014 [13]). Therefore, we explored the range of 60-100% for the multi-way sensitivity analyses. | | n/a | 71-91% one-way  60-100% multi-way |
| **Postpartum women retained in care and virologically suppressed at 12 months in MCH-ART** | | | | |
| 67% | | Myer et al. *PLoS Med* 2018 [1] – MCH-ART trial: 67% of women in the MCH-ART intervention were retained and virologically suppressed at 12 months postpartum, compared with 49% of women in the SOC arm.  Most other studies in the literature reporting on VS among postpartum women in resource-limited settings only reported the % VS among those retained, and not on the overall % retained out of the starting cohort.  Chetty et al. *Trop Med Int Health* 2018 [14] – South Africa: 75.5% of women retained, VS = <50 copies/mL  Mancinelli et al. *AIDS Res Hum Retroviruses* 2016 [15] – Malawi: 73.3% of women retained, VS = <50 copies/mL  Koss et al. *JAIDS* 2017 [16] – Uganda: Median follow-up time was 4.2 years (not 12 months postpartum). VS (<400 copies/mL) among those in care was 89.6% (95% CI: 83.2-94.2%). Assigning those not in care to unsuppressed status resulted in VS of 60.5% (95% CI: 53.6-67.3%) of overall cohort. | n/a | 49-75% one-way  48-74% multi-way |
| **Breastfeeding duration, mean (SD)** | | | | |
| 6 (6) SOC  8 (6) MCH-ART | | Myer et al. *PLoS Med* 2018 [1] – MCH-ART trial: 6 months (SD: 6 months) in SOC, 8 months (SD: 6 months) in MCH-ART  Somé et al. *Int Breastfeed J* 2017 [17] – South Africa: Median (IQR) duration of any breastfeeding among mothers with HIV was 29.1 weeks (13.0, 46.3)  Flynn et al. *JAIDS* 2018 [18] - India, Malawi, South Africa, Tanzania, Uganda, Zambia, and Zimbabwe: Median duration of breastfeeding was 16 months. | 0-16 months | 0-18 months |
| **Exclusive breastfeeding, %** | | | | |
| 71% SOC  77% MCH-ART | | Myer et al. *PLoS Med* 2018 [1] – MCH-ART trial: 71% in SOC, 77% in MCH-ART. Other studies from sub-Saharan Africa that reported on exclusive breastfeeding among mothers with HIV:  Somé et al. *Int Breastfeed J* 2017 [17] – Burkina Faso, South Africa, Uganda, and Zambia: 75.3% at 5 months  Bork et al. *JAIDS* 2013 [19] – Burkino Faso, Kenya, and South Africa: 22% at 5 months | 22-77% | 0-100% |
| **Probability of infant having a 6-10 week HIV test, %** | | | | |
| 78% SOC  82% MCH-ART | | Myer et al. *PLoS Med* 2018 [1] – MCH-ART trial: 78% in SOC, 82% in MCH-ART  Sherman et al. *J Glob Health* 2017 [20] – South Africa National Health Laboratory Service estimate for 2014: 87% EID coverage. | 78-87% | 0-100% |
| **Probability of infant having an HIV test at 18 months of age, %** | | | | |
| 71% SOC  81% MCH-ART | | Myer et al. *PLoS Med* 2018 [1] – MCH-ART trial did not report the uptake of late EID testing, as many infants may have had rapid tests that are not reported in the National Health Laboratory Service system, and Road to Health cards were not reliably completed. Therefore, we made the assumption that 18 month EID uptake resembled 12-month maternal retention in care in each strategy, and we varied late EID testing widely in sensitivity analyses. | n/a | 0-100% |

| **Mother-to-child transmission risks** | | | |
| --- | --- | --- | --- |
| ***In utero*/*intra partum* transmission, on ART, virologically suppressed** | | | |
| 0.44%, one-time risk | Point estimate derived from pooled values, weighted by eligible mother-infant pairs analysed in each study. Reviews of PMTCT trials that reported on virologic suppression at delivery for women initiated on 3-drug ART during the index pregnancy:  Mandelbrot et al. *Clin Infect Dis* 2015 [21]: 0.38-0.52% (VS = VL <50 copies/mL or undetectable, but with VL >50 copies/mL threshold)  Myer et al. *HIV Med* 2017 [2]: 0.25% (VS = VL <50 copies/mL)  Shapiro et al. *NEJM* 2010 [3]: 0.48% (VS = VL <50 copies/mL)  Perry et al. *HIV Med* 2016 [22]: 0.41% (VS = VL <50 copies/mL)  Kesho Bora Study Group *Lancet Infect Dis* 2011 [4]: 0.00% (VS = VL <300 copies/mL)  Cohan et al. *AIDS* 2015 [5]: 0.31% (VS = VL <400 copies/mL) | 0.00-0.48% | 0.00-0.88% |
| ***In utero*/*intra partum* transmission, on ART, not virologically suppressed** | | | |
| 2.57%, one-time risk | Point estimate derived from pooled values, weighted by eligible mother-infant pairs analysed in each study. Reviews of PMTCT trials that reported on virologic suppression at delivery for women initiated on 3-drug ART during the index pregnancy:  Mandelbrot et al. *Clin Infect Dis* 2015 [21]: 1.95% (VL 50-400 copies/mL)  Mandelbrot et al. *Clin Infect Dis* 2015 [21]: 3.06% (VL >400 copies/mL)  Myer et al. *HIV Med* 2017 [2]: 1.96% (VL 50-100 copies/mL)  Myer et al. *HIV Med* 2017 [2]: 8.51% (VL >1000 copies/mL)  Shapiro et al. *NEJM* 2010 [3]: 1.44% (VL >50 copies/mL)  Perry et al. *HIV Med* 2016 [22]: 1.37% (VL >50 copies/mL)  Kesho Bora Study Group *Lancet Infect Dis* 2011[4]: 5.34% (VL >300 copies/mL)  Cohan et al. *AIDS* 2015 [5]: 0.00% (VL >400 copies/mL) | 0.00-8.51% | 0.00-10.00% |
| ***Postpartum* transmission, on ART, virologically suppressed** | | | |
| 0.05%/mo. | Point estimate derived from pooled values, weighted by eligible mother-infant pairs analysed in each study. Included studies reported virologic suppression rates postpartum for cohorts of women who had initiated 3-drug ART during the current pregnancy and were continued on ART throughout breastfeeding. Overall postpartum MTCT risks were divided by the mean (or median) breastfeeding duration reported in each study to estimate the monthly risk of MTCT.  Shapiro et al. *NEJM* 2010 [3]: 0.10%/mo.  Cohan et al. *AIDS* 2015 [5]: 0.04%/mo.  Ngoma et al. *J Int AIDS Soc* 2015 [23] 0.00%/mo.  Peltier et al. *AIDS* 2009 [24] 0.00%/mo. | 0.00-0.10% | 0.00-0.10% |
| ***Postpartum* transmission, on ART, not virologically suppressed** | | | |
| 0.21%/mo. | Point estimate derived from pooled values, weighted by eligible mother-infant pairs analysed in each study. Included studies reported virologic suppression rates postpartum for cohorts of women who had initiated 3-drug ART during the current pregnancy and were continued on ART throughout breastfeeding. Overall postpartum MTCT risks were divided by the mean (or median) breastfeeding duration reported in each study to estimate the monthly risk of MTCT.  Shapiro et al. *NEJM* 2010 [3]: 0.00%/mo.  Cohan et al. *AIDS* 2015 [5]: 0.00%/mo.  Ngoma et al. *J Int AIDS Soc* 2015 [23]: 1.19%/mo.  Peltier et al. *AIDS* 2009 [24]: 0.21%/mo.  Two studies reported on late ART initiation in pregnancy at >34 weeks gestation. As virologic suppression is unlikely to be achieved in the early postpartum period with very late ART initiation, early postpartum MTCT from these cohorts were also included in the not virologically suppressed category:  Kilewo et al. *J Acquir Immune Defic Syndr* 2008 [25]: 0.20%/mo.  Thomas et al. *PLoS Med* 2011 [26]: 0.19%/mo. | 0.00-1.19% | 0.00-1.20% |
| ***Postpartum* transmission, not on ART** | | | |
| CD4 <350  Exclusive BF: 0.76%/mo.  Mixed BF: 1.28%/mo. | Monthly probability of MTCT risk was stratified by CD4 and breastfeeding status based on the ZVITAMBO cohort.  EBF – Iliff et al. *AIDS* 2005 [27]: 0.76%/mo.  MBF – Iliff et al. *AIDS* 2005 [27]: 1.28%/mo.  Best-case: Leroy et al. *Lancet* 1998 [28]: Both EBF/MBF: 0.48%/mo.  Worst-case: Coutsoudis et al. *Lancet* 1999 [29]: EBF: 1.49%/mo.; MBF: 2.34%/mo. | 0.48-2.34% | EBF: 0.19-1.52%  MBF: 0.32-2.56% |
| CD4 >350  Exclusive BF: 0.24%/m  Mixed BF: 0.40%/m | As above.  EBF – Iliff et al. *AIDS* 2005 [27]: 0.24%/mo.  MBF – Iliff et al. *AIDS* 2005 [27]: 0.40%/mo.  Best-case: Leroy et al. *Lancet* 1998 [28]: Both EBF/MBF: 0.15%/mo.  Worst-case: Coutsoudis et al. *Lancet* 1999 [29]: EBF: 0.47%/mo.; MBF: 0.73%/mo. | 0.15-0.73% | EBF: 0.06-0.48%  MBF: 0.10-0.80% |
| **Costs (in 2016 USD)** | | | |
| **First 12-months postpartum healthcare costs in MCH-ART** | | | |
| SOC: $50  MCH-ART: $69 | Myer et al. *PLoS Med* 2018 [1] – First 12-month postpartum healthcare costs derived from dedicated MCH-ART costing study. With no other literature on costs of postpartum integrated care programs available, varied widely for sensitivity analyses (0.5x-2x). |  | $35-138 |
| **Maternal ART (per month)** | | | |
| 1st line: $9  2nd line: $27 | Clinton Health Access Initiative. Antiretroviral CHAI reference price list 2016 [cited 2017 September 27]. Available from: <https://clintonhealthaccess.org/content/uploads/2016/11/2016-CHAI-ARV-Reference-Price-List_FINAL.pdf>.[30] | n/a | 1st line: $5-$18  2nd line: $14-$54 |
| **Pediatric ART (per month, range by weight)** | | | |
| 1st line: $21-$44  2nd line: $10-$25 | Clinton Health Access Initiative. Antiretroviral CHAI reference price list 2016 [cited 2017 September 27]. Available from: <https://clintonhealthaccess.org/content/uploads/2016/11/2016-CHAI-ARV-Reference-Price-List_FINAL.pdf>. [30]  Doherty K, Essajee S, Penazzato M, Holmes C, Resch S, Ciaranello A. Estimating age-based antiretroviral therapy costs for HIV-infected children in resource-limited settings based on World Health Organization weight-based dosing recommendations. BMC Health Serv Res. 2014;14:201. [31] | n/a | 1st line: $11-$88  2nd line: $5-$50 |
| **HIV routine care costs (range by CD4, per month)** | | | |
| $17-129 | Holmes C, Wood R, Badri M. CD4 decline and incidence of opportunistic infections in Cape Town, South Africa: Implications for prophylaxis and treatment. *J Acquir Immune Defic Syndr.* 2006;42:464–9. [32]  Cleary S, Okorafor OA, Chitha W, Boulle A, Jikwana S. Financing antiretroviral treatment and primary health care services. *South African Heal Rev*. 2005;58–74. [33] | n/a | $9-$258 |
| **OI care, per event (range by age, CD4 T-cell %/count, type of event)** | | | |
| <5 years old: $808-$1420 | Cleary S, Chitha W, Jikwana S, Okorafor OA, Boulle A. Health systems trust: South African health review. 2005. [33]  Thomas LS. Costing of HIV/AIDS services at a tertiary level hospital in Gauteng Province: Faculty of Health Sciences, University of Witwatersrand, South Africa; 2006 [cited 2014 May 6]. Available from: <http://wiredspace.wits.ac.za/handle/10539/2008>. [34] | n/a | $404-$2840 |
| >5 years old: $202-$679 | Holmes C, Wood R, Badri M. CD4 decline and incidence of opportunistic infections in Cape Town, South Africa: Implications for prophylaxis and treatment. *J Acquir Immune Defic Syndr.* 2006;42:464–9. [32]  Cleary S, Okorafor OA, Chitha W, Boulle A, Jikwana S. Financing antiretroviral treatment and primary health care services. *South African Heal Rev*. 2005;58–74. [33] | n/a | $101-$1358 |

**References:**

1. Myer L, Phillips TK, Zerbe A, Brittain K, Lesosky M, Hsiao NY, et al. Integration of postpartum healthcare services for HIV-infected women and their infants in South Africa: A randomised controlled trial. PLoS Med. 2018;15(3):e1002547.

2. Myer L, Phillips TK, McIntyre JA, Hsiao NY, Petro G, Zerbe A, et al. HIV viraemia and mother-to-child transmission risk after antiretroviral therapy initiation in pregnancy in Cape Town, South Africa. HIV Med. 2017;18(2):80-8.

3. Shapiro RL, Hughes MD, Ogwu A, Kitch D, Lockman S, Moffat C, et al. Antiretroviral regimens in pregnancy and breast-feeding in Botswana. N Engl J Med. 2010;362(24):2282-94.

4. Kesho Bora Study Group. Triple antiretroviral compared with zidovudine and single-dose nevirapine prophylaxis during pregnancy and breastfeeding for prevention of mother-to-child transmission of HIV-1 (Kesho Bora study): a randomised controlled trial. Lancet Infect Dis. 2011;11(3):171-80.

5. Cohan D, Natureeba P, Koss CA, Plenty A, Luwedde F, Mwesigwa J, et al. Efficacy and safety of lopinavir/ritonavir versus efavirenz-based antiretroviral therapy in HIV-infected pregnant Ugandan women. AIDS. 2015;29(2):183-91.

6. Fowler MG, Qin M, Fiscus SA, Currier JS, Flynn PM, Chipato T, et al. Benefits and Risks of Antiretroviral Therapy for Perinatal HIV Prevention. N Engl J Med. 2016;375(18):1726-37.

7. Chagomerana MB, Miller WC, Tang JH, Hoffman IF, Mthiko BC, Phulusa J, et al. Optimizing prevention of HIV mother to child transmission: Duration of antiretroviral therapy and viral suppression at delivery among pregnant Malawian women. PLoS One. 2018;13(4):e0195033.

8. Rotheram-Borus MJ, Tomlinson M, Scheffler A, Le Roux IM. Re-engagement in HIV care among mothers living with HIV in South Africa over 36 months post-birth. AIDS. 2015;29(17):2361-2.

9. Kaplan SR, Oosthuizen C, Stinson K, Little F, Euvrard J, Schomaker M, et al. Contemporary disengagement from antiretroviral therapy in Khayelitsha, South Africa: A cohort study. PLoS Med. 2017;14(11):e1002407.

10. Bershetyn A, Odeny TA, Lyamuya R, Nakiwogga-Muwanga A, Diero L, Bwana M, et al. The Causal Effect of Tracing by Peer Health Workers on Return to Clinic Among Patients Who Were Lost to Follow-up From Antiretroviral Therapy in Eastern Africa: A "Natural Experiment" Arising From Surveillance of Lost Patients. Clin Infect Dis. 2017;64(11):1547-54.

11. Knettel BA, Cichowitz C, Ngocho JS, Knippler ET, Chumba LN, Mmbaga BT, et al. Retention in HIV care during pregnancy and the postpartum period in the option B+ era: A systematic review and meta-analysis of studies in Africa. J Acquir Immune Defic Syndr. 2017.

12. Llenas-Garcia J, Wikman-Jorgensen P, Hobbins M, Mussa MA, Ehmer J, Keiser O, et al. Retention in care of HIV-infected pregnant and lactating women starting ART under Option B+ in rural Mozambique. Trop Med Int Health. 2016;21(8):1003-12.

13. Kamuyango AA, Hirschhorn LR, Wang W, Jansen P, Hoffman RM. One-year outcomes of women started on antiretroviral therapy during pregnancy before and after the implementation of Option B+ in Malawi: A retrospective chart review. World J AIDS. 2014;4(3):332-7.

14. Chetty T, Newell ML, Thorne C, Coutsoudis A. Viraemia before, during and after pregnancy in HIV-infected women on antiretroviral therapy in rural KwaZulu-Natal, South Africa, 2010-2015. Trop Med Int Health. 2018;23(1):79-91.

15. Mancinelli S, Galluzzo CM, Andreotti M, Liotta G, Jere H, Sagno JB, et al. Virological response and drug resistance 1 and 2 years post-partum in HIV-infected women initiated on life-long antiretroviral therapy in Malawi. AIDS Res Hum Retroviruses. 2016;32(8):737-42.

16. Koss CA, Natureeba P, Kwarisiima D, Ogena M, Clark TD, Olwoch P, et al. Viral suppression and retention in care up to 5 years after initiation of lifelong ART during pregnancy (Option B+) in rural Uganda. J Acquir Immune Defic Syndr. 2017;74(3):279-84.

17. Some EN, Engebretsen IMS, Nagot N, Meda N, Lombard C, Vallo R, et al. Breastfeeding patterns and its determinants among mothers living with Human Immuno-deficiency Virus -1 in four African countries participating in the ANRS 12174 trial. Int Breastfeed J. 2016;12:22.

18. Flynn PM, Taha TE, Cababasay M, Fowler MG, Mofenson LM, Owor M, et al. Prevention of HIV-1 Transmission Through Breastfeeding: Efficacy and Safety of Maternal Antiretroviral Therapy Versus Infant Nevirapine Prophylaxis for Duration of Breastfeeding in HIV-1-Infected Women With High CD4 Cell Count (IMPAACT PROMISE): A Randomized, Open-Label, Clinical Trial. J Acquir Immune Defic Syndr. 2018;77(4):383-92.

19. Bork K, Cames C, Cournil A, Musyoka F, Ayassou K, Naidu K, et al. Infant feeding modes and determinants among HIV-1-infected African Women in the Kesho Bora Study. J Acquir Immune Defic Syndr. 2013;62(1):109-18.

20. Sherman GG, Mazanderani AH, Barron P, Bhardwaj S, Niit R, Okobi M, et al. Toward elimination of mother-to-child transmission of HIV in South Africa: how best to monitor early infant infections within the Prevention of Mother-to-Child Transmission Program. J Glob Health. 2017;7(1):010701.

21. Mandelbrot L, Tubiana R, Le Chenadec J, Dollfus C, Faye A, Pannier E, et al. No perinatal HIV-1 transmission from women with effective antiretroviral therapy starting before conception. Clin Infect Dis. 2015;61(11):1715-25.

22. Perry ME, Taylor GP, Sabin CA, Conway K, Flanagan S, Dwyer E, et al. Lopinavir and atazanavir in pregnancy: comparable infant outcomes, virological efficacies and preterm delivery rates. HIV Med. 2016;17(1):28-35.

23. Ngoma MS, Misir A, Mutale W, Rampakakis E, Sampalis JS, Elong A, et al. Efficacy of WHO recommendation for continued breastfeeding and maternal cART for prevention of perinatal and postnatal HIV transmission in Zambia. J Int AIDS Soc. 2015;18:19352.

24. Peltier CA, Ndayisaba GF, Lepage P, van Griensven J, Leroy V, Pharm CO, et al. Breastfeeding with maternal antiretroviral therapy or formula feeding to prevent HIV postnatal mother-to-child transmission in Rwanda. AIDS. 2009;23(18):2415-23.

25. Kilewo C, Karlsson K, Massawe A, Lyamuya E, Swai A, Mhalu F, et al. Prevention of mother-to-child transmission of HIV-1 through breast-feeding by treating infants prophylactically with lamivudine in Dar es Salaam, Tanzania: the Mitra Study. J Acquir Immune Defic Syndr. 2008;48(3):315-23.

26. Thomas TK, Masaba R, Borkowf CB, Ndivo R, Zeh C, Misore A, et al. Triple-antiretroviral prophylaxis to prevent mother-to-child HIV transmission through breastfeeding--the Kisumu Breastfeeding Study, Kenya: a clinical trial. PLoS Med. 2011;8(3):e1001015.

27. Iliff PJ, Piwoz EG, Tavengwa NV, Zunguza CD, Marinda ET, Nathoo KJ, et al. Early exclusive breastfeeding reduces the risk of postnatal HIV-1 transmission and increases HIV-free survival. AIDS. 2005;19(7):699-708.

28. Leroy V, Newell ML, Dabis F, Peckham C, Van de Perre P, Bulterys M, et al. International multicentre pooled analysis of late postnatal mother-to-child transmission of HIV-1 infection. Ghent International Working Group on Mother-to-Child Transmission of HIV. Lancet. 1998;352(9128):597-600.

29. Coutsoudis A, Pillay K, Spooner E, Kuhn L, Coovadia HM. Influence of infant-feeding patterns on early mother-to-child transmission of HIV-1 in Durban, South Africa: a prospective cohort study. South African Vitamin A Study Group. Lancet. 1999;354(9177):471-6.

30. Clinton Health Access Initiative. Antiretroviral CHAI reference price list <https://clintonhealthaccess.org/content/uploads/2016/11/2016-CHAI-ARV-Reference-Price-List_FINAL.pdf2016> [cited 2017 September 27]. Available from: <https://clintonhealthaccess.org/content/uploads/2016/11/2016-CHAI-ARV-Reference-Price-List_FINAL.pdf>.

31. Doherty K, Essajee S, Penazzato M, Holmes C, Resch S, Ciaranello A. Estimating age-based antiretroviral therapy costs for HIV-infected children in resource-limited settings based on World Health Organization weight-based dosing recommendations. BMC Health Serv Res. 2014;14:201.

32. Holmes CB, Wood R, Badri M, Zilber S, Wang B, Maartens G, et al. CD4 decline and incidence of opportunistic infections in Cape Town, South Africa: implications for prophylaxis and treatment. J Acquir Immune Defic Syndr. 2006;42(4):464-9.

33. Cleary S, Chitha W, Jikwana S, Okorafor OA, Boulle A. Health systems trust: South African health review. 2005.

34. Thomas LS. Costing of HIV/AIDS services at a tertiary level hospital in Gauteng Province: Faculty of Health Sciences, University of Witwatersrand, South Africa; 2006 [cited 2014 May 6]. Available from: <http://wiredspace.wits.ac.za/handle/10539/2008>.
