# Supplementary material for: Cost-effectiveness of integrating postpartum antiretroviral therapy and infant care into maternal & child health services in South Africa
Source: PLoS One. 2019 Nov 15;14(11):e0225104. doi: 10.1371/journal.pone.0225104 (PMC6857940; doi:10.1371/journal.pone.0225104)
Supplement: S3 Table — (DOCX) [file pone.0225104.s008.docx]

**S3 Table. Model output used to determine the cost-effectiveness threshold**

| **Strategy** |  | **Maternal^a^** | | **Pediatric^a^** | | **Maternal + Pediatric^a^** | | **ICER ($/year of life saved)** |
| --- | --- | --- | --- | --- | --- | --- | --- | --- |
|  |  | **LE (years)** | **Lifetime cost (US $)** | **LE**  **(years)** | **Lifetime cost (US $)** | **LE**  **(years)** | **Lifetime cost (US $)** |  |
| **CET scenario** | |  | | | | | | |
| *First-line ART only* |  | 18.77 (12.47) | 10,300  (6,920) | 62.03  (26.02) | 229  (174) | 80.80  (38.49) | 10,529  (7,094) | *Comparator* |
| *Second-line ART available* |  | 25.26 (15.98) | 16,329 (10,122) | 62.23  (26.09) | 347  (212) | 87.49  (42.07) | 16,676  (10,334) | 903 |
| ***CET:*** *cost-effectiveness threshold;* ***LE:*** *Life expectancy;* ***ICER:*** *Incremental cost-effectiveness ratio* | | | | | | | | |
| **^a.^** All costs are reported in 2016 US$. Maternal life expectancy was projected from delivery and pediatric life expectancy was projected from birth. Pediatric cost and life expectancy results reflect the entire cohort of HIV-exposed children, both HIV-infected and HIV-uninfected. Life expectancy and costs were discounted at a rate of 3% and shown in parentheses. Undiscounted life expectancy and cost projections are shown without parentheses. ICERs were calculated from discounted values prior to rounding.  **^b.^** The ICER of second-line ART relative to first-line ART only for mothers alone, without pediatric outcomes was $912/YLS. | | | | | | | | |
|  | | | | | | | | |
