# Supplementary material for: Cost-effectiveness of integrating postpartum antiretroviral therapy and infant care into maternal & child health services in South Africa
Source: PLoS One. 2019 Nov 15;14(11):e0225104. doi: 10.1371/journal.pone.0225104 (PMC6857940; doi:10.1371/journal.pone.0225104)
Supplement: S4 Table — (DOCX) [file pone.0225104.s009.docx]

|  |
| --- |

**S4 Table. Calibration and validation of maternal engagement in care.**

|  | **Retention targets** | | **Suppression (VS) targets** | |
| --- | --- | --- | --- | --- |
|  | **Alive and retained in care at 1 year,**  **% (95% CI)^a,c^** | **Alive and retained in care at 3 years,**  **% (95% CI)^b,c^** | **VS of those alive**  **at 1 year,**  **% (95% CI)^a,c^** | **VS of those alive and in care at 3 years,**  **% (95% CI)^b,c^** |
| **Standard of care** | | | | |
| *External target* | 70.59 (64.36-76.30) | 69.70 (69.09-70.22) | 49.37 (42.83-55.92) | 77.62 (74.75-80.30) |
| *Model result* | 70.98 | 69.65 | 49.12 | 75.58 |
| **MCH-ART** | | | | |
| *External target* | 80.69 (75.02-85.55) | N/A^c^ | 66.81 (60.35-72.84) | N/A^c^ |
| *Model result* | 81.27 | 76.40 | 66.41 | 76.09 |
| ^a^ For both strategies, model inputs for ART adherence were calibrated to hit 1-year retention and virologic suppression targets from the MCH-ART trial, and then partially dependently externally validated by comparing model output to MCH-ART trial data [1].  ^b^ Standard of care 3-year calibration and validation targets for retention in care and virologic suppression were taken from the published literature [2-5]. After 1 year, individuals in the MCH-ART strategy received the same monthly probabilities of loss to follow-up and virologic failure as were calibrated for the standard of care strategy.  ^c^ All model results were within 95% confidence limits of the external targets that were drawn from the MCH-ART trial and published literature. We calculated exact binomial confidence limits using the Clopper-Pearson method. The 95% confidence interval for 3-year virologic suppression was calculated based off of data from three different studies, all weighted by the number of subjects contributing data in each analysis. | | | | |

**REFERENCES:**

1. Myer L, Phillips TK, Zerbe A, Brittain K, Lesosky M, Hsiao NY, et al. Integration of postpartum healthcare services for HIV-infected women and their infants in South Africa: A randomised controlled trial. PLoS Med. 2018;15(3):e1002547.

2. Koss CA, Natureeba P, Kwarisiima D, Ogena M, Clark TD, Olwoch P, et al. Viral suppression and retention in care up to 5 years after initiation of lifelong ART during pregnancy (Option B+) in rural Uganda. J Acquir Immune Defic Syndr. 2017;74(3):279-84.

3. Mancinelli S, Galluzzo CM, Andreotti M, Liotta G, Jere H, Sagno JB, et al. Virological response and drug resistance 1 and 2 years post-partum in HIV-infected women initiated on life-long antiretroviral therapy in Malawi. AIDS Res Hum Retroviruses. 2016;32(8):737-42.

4. Haas AD, Tenthani L, Msukwa MT, Tal K, Jahn A, Gadabu OJ, et al. Retention in care during the first 3 years of antiretroviral therapy for women in Malawi's option B+ programme: an observational cohort study. Lancet HIV. 2016;3(4):e175-82.

5. Chetty T, Newell ML, Thorne C, Coutsoudis A. Viraemia before, during and after pregnancy in HIV-infected women on antiretroviral therapy in rural KwaZulu-Natal, South Africa, 2010-2015. Trop Med Int Health. 2018;23(1):79-91.
