# Supplementary material for: Cost-effectiveness of integrating postpartum antiretroviral therapy and infant care into maternal & child health services in South Africa
Source: PLoS One. 2019 Nov 15;14(11):e0225104. doi: 10.1371/journal.pone.0225104 (PMC6857940; doi:10.1371/journal.pone.0225104)
Supplement: S5 Table — (DOCX) [file pone.0225104.s010.docx]

**S5 Table. Results of selected one-way sensitivity analyses**

|  |  |  | **Clinical Outcomes** | | | | **Undiscounted model results** | | | | | | **Discounted model results** | | |
| --- | --- | --- | --- | --- | --- | --- | --- | --- | --- | --- | --- | --- | --- | --- | --- |
|  |  |  | Maternal 1-year mortality (%) | Pediatric HIV infection (%) |  | LE of HIV-infected infants (years) | **Maternal** | | **Pediatric** | | **Combined Maternal + Pediatric** | | **Combined Maternal + Pediatric** | | |
|  |  |  |  |  | Pediatric 1-year mortality (%) |  | LE from delivery (years) | HIV-related costs/ person | LE (years) | HIV-related costs/ person | LE from delivery (years) | HIV-related costs/ pair | LE from delivery (years) | HIV-related costs/ pair | ICER ($/YLS) |
| **I. Clinical parameters** | | | | | | | | | | | | | | | |
| Base case | | |  |  |  |  |  |  |  |  |  |  |  |  |  |
| *SOC* | | | 1.7 | 2.08 | 6.3 | 23.13 | 25.26 | 16,119 | 62.23 | 346 | 87.49 | 16,515 | 42.07 | 10,173 | *Comparator* |
| *MCH-ART* | | | 1.6 | 2.06 | 6.2 | 23.40 | 26.20 | 16,697 | 62.25 | 346 | 88.45 | 17,112 | 42.60 | 10,487 | **599** |
| Maternal pre-ART CD4 count, median | | |  |  |  |  |  |  |  |  |  |  |  |  |  |
| Decreased to 250 cells/µL | | |  |  |  |  |  |  |  |  |  |  |  |  |  |
| *SOC* | | | 1.7 | 2.16 | 6.3 | 23.25 | 23.90 | 15,708 | 62.21 | 358 | 86.11 | 16,117 | 41.40 | 10,137 | *Comparator* |
| *MCH-ART* | | | 1.6 | 2.12 | 6.3 | 23.38 | 24.92 | 16,333 | 62.23 | 356 | 87.15 | 16,760 | 41.99 | 10,469 | **560** |
| Increased to 550 cells/µL | | |  |  |  |  |  |  |  |  |  |  |  |  |  |
| *SOC* | | | 1.3 | 1.87 | 6.2 | 23.12 | 28.16 | 17,023 | 62.33 | 315 | 90.49 | 17,379 | 43.64 | 10,266 | *Comparator* |
| *MCH-ART* | | | 1.2 | 1.87 | 6.2 | 23.16 | 28.95 | 17,496 | 62.34 | 317 | 91.29 | 17,869 | 44.03 | 10,520 | **643** |
| Mothers retained and virologically suppressed in MCH-ART (retention constant at 81%) | | |  |  |  |  |  |  |  |  |  |  |  |  |  |
|  | *SOC (49%)* | | 1.7 | 2.08 | 6.3 | 23.13 | 25.26 | 16,119 | 62.23 | 346 | 87.49 | 16,515 | 42.07 | 10,173 | *Comparator* |
|  | *MCH-ART* | |  |  |  |  |  |  |  |  |  |  |  |  |  |
|  |  | Decreased to 49% | 1.6 | 2.17 | 6.3 | 23.51 | 25.69 | 16,701 | 62.22 | 365 | 87.91 | 17,136 | 42.36 | 10,607 | **1,525** |
|  |  | Decreased to 55% | 1.6 | 2.16 | 6.3 | 23.48 | 25.85 | 16,636 | 62.22 | 363 | 88.07 | 17,069 | 42.42 | 10,530 | **1,006** |
|  |  | Decreased to 60% | 1.6 | 2.11 | 6.2 | 23.41 | 26.01 | 16,660 | 62.24 | 355 | 88.25 | 17,084 | 42.51 | 10,504 | **763** |
|  |  | Decreased to 65% | 1.6 | 2.06 | 6.2 | 23.34 | 26.13 | 16,682 | 62.26 | 347 | 88.39 | 17,098 | 42.56 | 10,489 | **637** |
|  |  | Increased to 70% | 1.6 | 2.00 | 6.2 | 23.23 | 26.24 | 16,754 | 62.28 | 336 | 88.52 | 17,159 | 42.64 | 10,499 | **576** |
|  |  | Increased to 75% | 1.6 | 1.99 | 6.2 | 23.22 | 26.35 | 16,720 | 62.29 | 335 | 88.64 | 17,123 | 42.69 | 10,455 | **458** |
| Maternal 12-month retention in MCH-ART | | |  |  |  |  |  |  |  |  |  |  |  |  |  |
|  | *SOC* | | 1.7 | 2.08 | 6.3 | 23.13 | 25.26 | 16,119 | 62.23 | 346 | 87.49 | 16,515 | 42.07 | 10,173 | *Comparator* |
|  | *MCH-ART* | |  |  |  |  |  |  |  |  |  |  |  |  |  |
|  |  | Decreased to 71% | 1.7 | 2.35 | 6.3 | 23.59 | 25.42 | 16,169 | 62.14 | 391 | 87.56 | 16,626 | 42.14 | 10,196 | **386** |
|  |  | Increased to 91% | 1.4 | 1.76 | 6.2 | 22.92 | 26.91 | 17,193 | 62.38 | 299 | 89.29 | 17,564 | 43.04 | 10,758 | **604** |
| Duration of the intervention effect (12 months) | | |  |  |  |  |  |  |  |  |  |  |  |  |  |
|  | *SOC* | | 1.7 | 2.08 | 6.3 | 23.13 | 25.26 | 16,119 | 62.23 | 346 | 87.49 | 16,515 | 42.07 | 10,173 | *Comparator* |
|  | *MCH-ART* | |  |  |  |  |  |  |  |  |  |  |  |  |  |
|  |  | 0.5x base case (6 months) | 1.7 | 2.20 | 6.3 | 23.58 | 25.77 | 16,513 | 62.20 | 371 | 87.97 | 16,944 | 42.35 | 10,428 | **889** |
|  |  | 1.5x base case (18 months) | 1.5 | 1.97 | 6.2 | 23.42 | 26.50 | 16,813 | 62.29 | 334 | 88.79 | 17,225 | 42.77 | 10,521 | **500** |
| Maternal background return to care probability (base case = 1.3%/month) | | |  |  |  |  |  |  |  |  |  |  |  |  |  |
|  | 0% per month | |  |  |  |  |  |  |  |  |  |  |  |  |  |
|  |  | *SOC* | 1.8 | 2.08 | 6.3 | 23.13 | 20.95 | 12,348 | 62.23 | 346 | 83.18 | 12,744 | 40.11 | 8,318 | *Comparator* |
|  |  | *MCH-ART* | 1.6 | 2.06 | 6.2 | 23.40 | 22.19 | 13,116 | 62.25 | 346 | 84.44 | 13,531 | 40.83 | 8,779 | **639** |
|  | 5% per month | |  |  |  |  |  |  |  |  |  |  |  |  |  |
|  |  | *SOC* | 1.7 | 2.08 | 6.3 | 23.13 | 28.62 | 19,558 | 62.23 | 346 | 90.85 | 19,954 | 43.67 | 11,913 | *Comparator* |
|  |  | *MCH-ART* | 1.6 | 2.06 | 6.2 | 23.40 | 29.28 | 19,910 | 62.25 | 346 | 91.53 | 20,325 | 44.02 | 12,063 | **433** |
| **I. Clinical parameters (continued)** | | | | | | | | | | | | | | | |
| Maternal LTFU rates after 12 months (base case = 0.3-0.6%/month, range by ART adherence) | | |  |  |  |  |  |  |  |  |  |  |  |  |  |
|  | 0.5x base case | |  |  |  |  |  |  |  |  |  |  |  |  |  |
|  |  | *SOC* | 1.7 | 2.08 | 6.3 | 23.13 | 27.00 | 18,103 | 62.23 | 346 | 89.23 | 18,499 | 42.84 | 11,130 | *Comparator* |
|  |  | *MCH-ART* | 1.6 | 2.06 | 6.2 | 23.40 | 28.05 | 18,822 | 62.25 | 346 | 90.30 | 19,237 | 43.42 | 11,507 | **655** |
|  | 2x base case | |  |  |  |  |  |  |  |  |  |  |  |  |  |
|  |  | *SOC* | 1.7 | 2.08 | 6.3 | 23.13 | 22.60 | 13,446 | 62.23 | 346 | 84.83 | 13,842 | 40.87 | 8,846 | *Comparator* |
|  |  | *MCH-ART* | 1.6 | 2.06 | 6.2 | 23.40 | 23.37 | 13,857 | 62.25 | 346 | 85.62 | 14,272 | 41.32 | 9,081 | **522** |
| Relative risk reduction in chronic AIDS mortality while on ART (adults) | | |  |  |  |  |  |  |  |  |  |  |  |  |  |
|  | 0% reduction | |  |  |  |  |  |  |  |  |  |  |  |  |  |
|  |  | *SOC* | 3.6 | 2.08 | 6.3 | 18.86 | 18.33 | 10,881 | 62.15 | 274 | 80.48 | 11,205 | 38.64 | 7,570 | *Comparator* |
|  |  | *MCH-ART* | 3.6 | 2.06 | 6.2 | 18.94 | 18.97 | 11,272 | 62.17 | 274 | 81.14 | 11,615 | 39.02 | 7,819 | **645** |
|  | 100% reduction | |  |  |  |  |  |  |  |  |  |  |  |  |  |
|  |  | *SOC* | 1.7 | 2.08 | 6.3 | 26.57 | 27.65 | 21,072 | 62.31 | 494 | 89.96 | 21,615 | 43.09 | 12,219 | *Comparator* |
|  |  | *MCH-ART* | 1.5 | 2.06 | 6.2 | 26.72 | 28.57 | 21,586 | 62.33 | 493 | 90.90 | 22,148 | 43.59 | 12,458 | **486** |
| Mean duration of breastfeeding, months | | |  |  |  |  |  |  |  |  |  |  |  |  |  |
|  | 6 months in both strategies | |  |  |  |  |  |  |  |  |  |  |  |  |  |
|  |  | *SOC* | 1.7 | 2.08 | 6.3 | 23.13 | 25.26 | 16,119 | 62.23 | 346 | 87.49 | 16,515 | 42.07 | 10,173 | *Comparator* |
|  |  | *MCH-ART* | 1.6 | 1.79 | 6.2 | 23.17 | 26.20 | 16,697 | 62.36 | 304 | 88.56 | 17,071 | 42.64 | 10,465 | **519** |
|  | 12 months in both strategies | |  |  |  |  |  |  |  |  |  |  |  |  |  |
|  |  | *SOC* | 1.7 | 3.42 | 6.4 | 24.47 | 25.26 | 16,119 | 61.72 | 566 | 86.98 | 16,735 | 41.91 | 10,292 | *Comparator* |
|  |  | *MCH-ART* | 1.6 | 2.70 | 6.3 | 24.26 | 26.20 | 16697 | 62.00 | 456 | 88.20 | 17,222 | 42.53 | 10,546 | **416** |
|  | 18 months in both strategies | |  |  |  |  |  |  |  |  |  |  |  |  |  |
|  |  | *SOC* | 1.7 | 5.26 | 6.4 | 25.58 | 25.26 | 16,119 | 61.01 | 881 | 86.27 | 17,050 | 41.70 | 10,460 | *Comparator* |
|  |  | *MCH-ART* | 1.6 | 4.02 | 6.3 | 25.44 | 26.20 | 16,697 | 61.51 | 681 | 87.71 | 17,447 | 42.38 | 10,666 | **306** |
| EID uptake in both strategies | | |  |  |  |  |  |  |  |  |  |  |  |  |  |
| 0% birth uptake | | |  |  |  |  |  |  |  |  |  |  |  |  |  |
| *SOC* | | | 1.7 | 2.08 | 6.3 | 22.53 | 25.26 | 16,119 | 62.23 | 319 | 87.49 | 16,488 | 42.07 | 10,150 | *Comparator* |
| *MCH-ART* | | | 1.6 | 2.06 | 6.2 | 22.80 | 26.20 | 16,697 | 62.23 | 320 | 88.43 | 17,086 | 42.59 | 10,469 | **603** |
| 100% birth uptake | | |  |  |  |  |  |  |  |  |  |  |  |  |  |
| *SOC* | | | 1.7 | 2.08 | 6.3 | 23.44 | 25.26 | 16,119 | 62.24 | 356 | 87.50 | 16,525 | 42.08 | 10,180 | *Comparator* |
| *MCH-ART* | | | 1.6 | 2.06 | 6.2 | 23.58 | 26.20 | 16,697 | 62.27 | 355 | 88.47 | 17,121 | 42.61 | 10,499 | **598** |
| 0% 6-10 week uptake | | |  |  |  |  |  |  |  |  |  |  |  |  |  |
| *SOC* | | | 1.7 | 2.08 | 6.3 | 21.97 | 25.26 | 16,119 | 62.21 | 311 | 87.47 | 16,481 | 42.06 | 10,140 | *Comparator* |
| *MCH-ART* | | | 1.6 | 2.06 | 6.2 | 22.08 | 26.20 | 16,697 | 62.23 | 308 | 88.43 | 17,074 | 42.59 | 10,459 | **595** |
| 100% 6-10 week uptake | | |  |  |  |  |  |  |  |  |  |  |  |  |  |
| *SOC* | | | 1.7 | 2.08 | 6.3 | 23.55 | 25.26 | 16,119 | 62.25 | 356 | 87.51 | 16,525 | 42.08 | 10,180 | *Comparator* |
| *MCH-ART* | | | 1.6 | 2.06 | 6.2 | 23.62 | 26.20 | 16,697 | 62.27 | 352 | 88.47 | 17,118 | 42.61 | 10,499 | **595** |
| 0% 18 month uptake | | |  |  |  |  |  |  |  |  |  |  |  |  |  |
| *SOC* | | | 1.7 | 2.08 | 6.3 | 22.10 | 25.26 | 16,119 | 62.22 | 329 | 87.48 | 16,498 | 42.07 | 10,160 | *Comparator* |
| *MCH-ART* | | | 1.6 | 2.06 | 6.2 | 22.12 | 26.20 | 16,697 | 62.23 | 326 | 88.43 | 17,092 | 42.59 | 10,479 | **599** |
| 100% 18 month uptake | | |  |  |  |  |  |  |  |  |  |  |  |  |  |
| *SOC* | | | 1.7 | 2.08 | 6.3 | 23.67 | 25.26 | 16,119 | 62.25 | 353 | 87.51 | 16,522 | 42.08 | 10,170 | *Comparator* |
| *MCH-ART* | | | 1.6 | 2.06 | 6.2 | 23.61 | 26.20 | 16,697 | 62.27 | 349 | 88.47 | 17,115 | 42.61 | 10,489 | **596** |
| **I. Clinical parameters (continued)** | | | | | | | | | | | | | | | |
| Relative risk of non-AIDS mortality with replacement feeding (RR-RF scenarios, see manuscript methods section) | | |  |  |  |  |  |  |  |  |  |  |  |  |  |
| RR-RF = 2 | | |  |  |  |  |  |  |  |  |  |  |  |  |  |
| *SOC* | | | 1.7 | 2.08 | 7.4 | 23.03 | 25.26 | 16,119 | 61.44 | 344 | 86.70 | 16,513 | 41.75 | 10,170 | *Comparator* |
| *MCH-ART* | | | 1.6 | 2.05 | 7.1 | 23.14 | 26.20 | 16,697 | 61.62 | 342 | 87.82 | 17,108 | 42.34 | 10,489 | **531** |
| RR-REF = 3 | | |  |  |  |  |  |  |  |  |  |  |  |  |  |
| *SOC* | | | 1.7 | 2.08 | 8.5 | 22.82 | 25.26 | 16,119 | 60.66 | 341 | 85.92 | 16,510 | 41.42 | 10,170 | *Comparator* |
| *MCH-ART* | | | 1.6 | 2.05 | 7.9 | 22.97 | 26.20 | 16,697 | 60.99 | 340 | 87.19 | 17,106 | 42.08 | 10,489 | **481** |
| **II. Cost parameters** | | | | | | | | | | | | | | | |
| Routine care costs (see S3 Table) | | |  |  |  |  |  |  |  |  |  |  |  |  |  |
|  | 0.5x base case | |  |  |  |  |  |  |  |  |  |  |  |  |  |
|  |  | *SOC* | 1.7 | 2.08 | 6.3 | 23.13 | 25.26 | 13,185 | 62.23 | 286 | 87.49 | 13,521 | 42.07 | 8,400 | *Comparator* |
|  |  | *MCH-ART* | 1.6 | 2.06 | 6.2 | 23.40 | 26.20 | 13,666 | 62.25 | 285 | 88.45 | 14,020 | 42.60 | 8,659 | **504** |
|  | 2x base case | |  |  |  |  |  |  |  |  |  |  |  |  |  |
|  |  | *SOC* | 1.7 | 2.08 | 6.3 | 23.13 | 25.26 | 21,984 | 62.23 | 466 | 87.49 | 22,500 | 42.07 | 13,730 | *Comparator* |
|  |  | *MCH-ART* | 1.6 | 2.06 | 6.2 | 23.40 | 26.20 | 22,771 | 62.25 | 463 | 88.45 | 23,303 | 42.60 | 14,139 | **785** |
| ART costs | | |  |  |  |  |  |  |  |  |  |  |  |  |  |
|  | 0.5x base case | |  |  |  |  |  |  |  |  |  |  |  |  |  |
|  |  | *SOC* | 1.7 | 2.08 | 6.3 | 23.13 | 25.26 | 14,087 | 62.23 | 311 | 87.49 | 14,448 | 42.07 | 8,890 | *Comparator* |
|  |  | *MCH-ART* | 1.6 | 2.06 | 6.2 | 23.40 | 26.20 | 14,551 | 62.25 | 310 | 88.45 | 14,930 | 42.60 | 9,129 | **463** |
|  | 2x base case | |  |  |  |  |  |  |  |  |  |  |  |  |  |
|  |  | *SOC* | 1.7 | 2.08 | 6.3 | 23.13 | 25.26 | 20,161 | 62.23 | 416 | 87.49 | 20,627 | 42.07 | 12,750 | *Comparator* |
|  |  | *MCH-ART* | 1.6 | 2.06 | 6.2 | 23.40 | 26.20 | 20,988 | 62.25 | 414 | 88.45 | 21,471 | 42.60 | 13,209 | **874** |
| 12-month cumulative postpartum healthcare costs in MCH-ART | | |  |  |  |  |  |  |  |  |  |  |  |  |  |
|  | *SOC ($50)* | | 1.7 | 2.08 | 6.3 | 23.13 | 25.26 | 16,119 | 62.23 | 346 | 87.49 | 16,515 | 42.07 | 10,173 | *Comparator* |
|  | *MCH-ART (base case = $69)* | |  |  |  |  |  |  |  |  |  |  |  |  |  |
|  |  | 0.5x base case ($35) | 1.6 | 2.06 | 6.2 | 23.40 | 26.20 | 16,697 | 62.25 | 346 | 88.45 | 17,078 | 42.60 | 10,455 | **533** |
|  |  | 2x base case ($138) | 1.6 | 2.06 | 6.2 | 23.40 | 26.20 | 16,697 | 62.25 | 346 | 88.45 | 17,181 | 42.60 | 10,558 | **730** |
| **^a.^** All costs are reported in 2016 US$. Maternal life expectancy was projected from delivery and pediatric life expectancy was projected from birth. Life expectancy and costs were discounted at a rate of 3%. ICERs were calculated from discounted values prior to rounding.  **^b.^** Combined maternal + pediatric costs also include 12-month cumulative postpartum healthcare costs for the mother-infant pair. 12-month postpartum care costs were not discounted, as they were incurred during the first year of the simulation. | | | | | | | | | | | | | | | |
